# CDH17 nanobodies facilitate rapid imaging of gastric cancer and efficient delivery of immunotoxin

**DOI:** 10.1186/s40824-022-00312-3

**Published:** 2022-11-26

**Authors:** Jingbo Ma, Xiaolong Xu, Chunjin Fu, Peng Xia, Ming Tian, Liuhai Zheng, Kun Chen, Xiaolian Liu, Yilei Li, Le Yu, Qinchang Zhu, Yangyang Yu, Rongrong Fan, Haibo Jiang, Zhifen Li, Chuanbin Yang, Chengchao Xu, Ying Long, Jigang Wang, Zhijie Li

**Affiliations:** 1 Department of Hyperbaric Medicine, Shenzhen People’s Hospital (The Second Clinical Medical College, Jinan University, The First Affiliated Hospital, Southern University of Science and Technology), Shenzhen, 518020 Guangdong P. R. China; 2 College of Traditional Chinese Medicine, Tianjin University of Traditional Chinese Medicine, Tianjin, 300193 P. R. China; 3 Department of Geriatrics, Shenzhen People’s Hospital (The Second Clinical Medical College, Jinan University, The First Affiliated Hospital, Southern University of Science and Technology), Shenzhen, 518020 Guangdong P. R. China; 4 Department of Hepatobiliary & Pancreatic Surgery, Zhongnan Hospital of Wuhan University, Wuhan, 430072 Hubei P. R. China; 5 Clinical Pharmacy Center, Nanfang Hospital, Southern Medical University, Guangzhou, 510515 Guangdong P. R. China; 6 Guangdong Provincial Key Laboratory of New Drug Screening, School of Pharmaceutical Sciences, Southern Medical University, Guangzhou, 510515 Guangdong P.R. China; 7 College of Pharmacy, Shenzhen Technology University, Shenzhen, 518118 P.R. China; 8 Health Science Center, Shenzhen University, Shenzhen, 518055 Guangdong P. R. China; 9 Deapartment of Biosciences and Nutrition, Karolinska Institute, 14157 Stockholm, Sweden; 10 Department of Chemistry, The University of Hong Kong, Pok Fu Lam, Hong Kong, P. R. China; 11 School of Chemistry and Chemical Engineering, Shanxi Datong University, Xing Yun Street, Pingcheng District, Datong, 037009 Shanxi P. R. China; 12 Artemisinin Research Center, and Institute of Chinese Materia Medica, China Academy of Chinese Medical Sciences, Beijing, 100700 P. R. China

**Keywords:** Nanobody, Cadherin-17, Immunotoxin, Gastric cancer, Targeted therapy, Jingbo Ma, Xiaolong Xu and Chunjin Fu are contribute equally to this work and share the first-authorship.

## Abstract

**Background:**

It is highly desirable to develop new therapeutic strategies for gastric cancer given the low survival rate despite improvement in the past decades. Cadherin 17 (CDH17) is a membrane protein highly expressed in cancers of digestive system. Nanobody represents a novel antibody format for cancer targeted imaging and drug delivery. Nanobody targeting CHD17 as an imaging probe and a delivery vehicle of toxin remains to be explored for its theragnostic potential in gastric cancer.

**Methods:**

Naïve nanobody phage library was screened against CDH17 Domain 1-3 and identified nanobodies were extensively characterized with various assays. Nanobodies labeled with imaging probe were tested in vitro and in vivo for gastric cancer detection. A CDH17 Nanobody fused with toxin PE38 was evaluated for gastric cancer inhibition in vitro and in vivo.

**Results:**

Two nanobodies (A1 and E8) against human CDH17 with high affinity and high specificity were successfully obtained. These nanobodies could specifically bind to CDH17 protein and CDH17-positive gastric cancer cells. E8 nanobody as a lead was extensively determined for tumor imaging and drug delivery. It could efficiently co-localize with CDH17-positive gastric cancer cells in zebrafish embryos and rapidly visualize the tumor mass in mice within 3 h when conjugated with imaging dyes. E8 nanobody fused with toxin PE38 showed excellent anti-tumor effect and remarkably improved the mice survival in cell-derived (CDX) and patient-derived xenograft (PDX) models. The immunotoxin also enhanced the anti-tumor effect of clinical drug 5-Fluorouracil.

**Conclusions:**

The study presents a novel imaging and drug delivery strategy by targeting CDH17. CDH17 nanobody-based immunotoxin is potentially a promising therapeutic modality for clinical translation against gastric cancer.

**Graphical Abstract:**

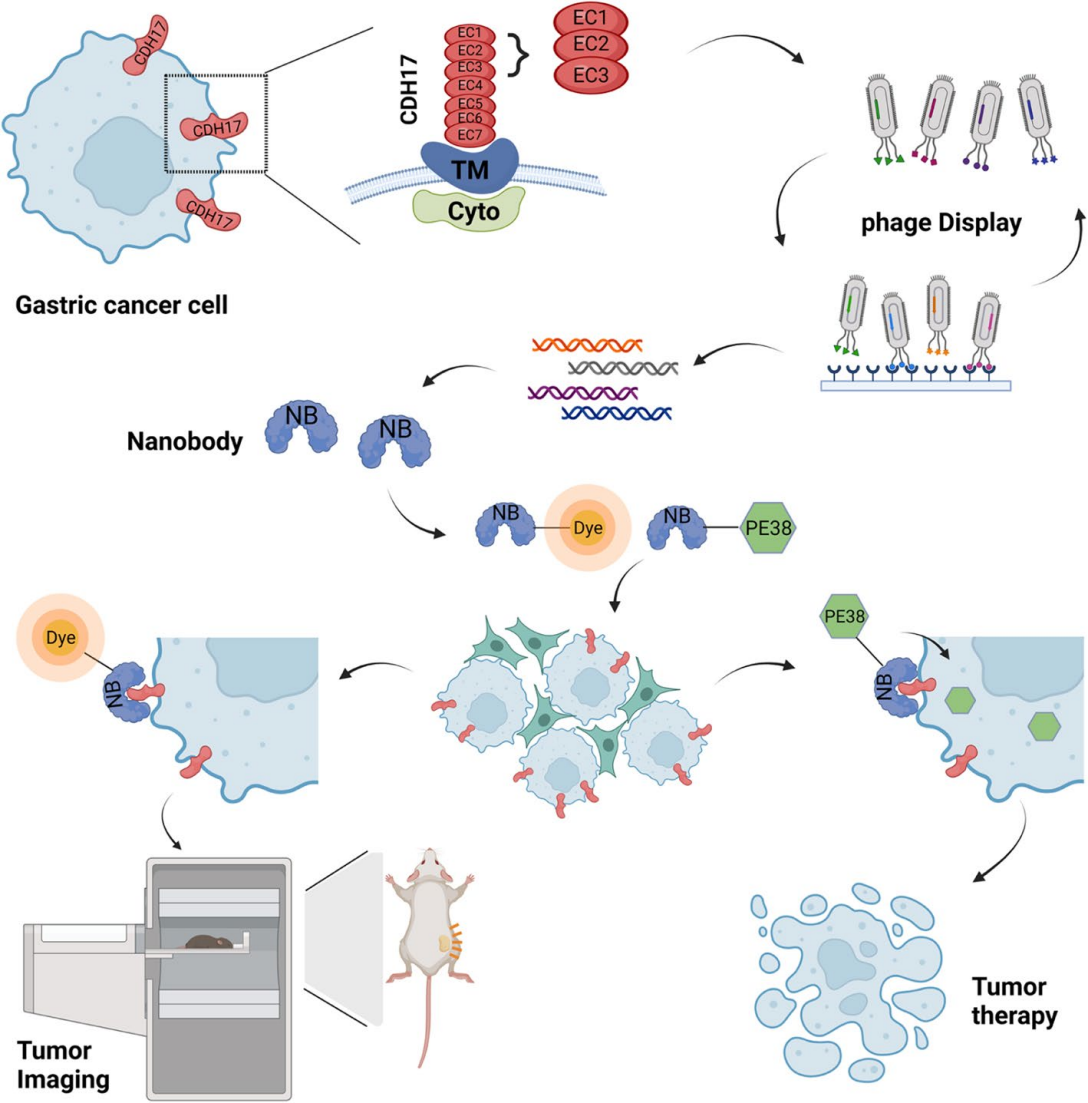

**Supplementary Information:**

The online version contains supplementary material available at 10.1186/s40824-022-00312-3.

## Introduction

Although the incidence and mortality of gastric cancer (GC) declined in the past decades, GC is still one of the most common malignancies and the third cause of cancer-related death worldwide [1]. Chemotherapy drugs remain the first-line treatment option for advanced GC (AGC). Only three targeted therapy drugs, namely HER2 antibody (Trastuzumab)/HER2-antibody drug conjugate(T-DXd), VEGFR2 antibody (ramucirumab) and PD-L1 antibody (Pembrolizumab), were approved for advanced and metastatic GC when used in combination with chemotherapy. Although these combination treatments improved overall survival (OS), the 5-year survival rate for AGCs remains less than 10% and the median OS is only approximately 1 year [2, 3]. Even if the recent combinatorial regimen of HER2 antibody plus PD-L1 antibody with chemotherapy showed favorable clinical results in unresectable and metastatic GC patients, the median duration of the response is just 1.1 months longer than the regimen without PD-L1 antibody [4, 5]. On the other hand, acquired drug resistance is commonly developed after the treatments with chemotherapy and/or HER2 antibody in GC patients [6, 7]. Thus, it is necessary and urgent to develop new strategies for advanced and metastatic GCs, especially design the new targeted drugs against novel molecules beyond HER2, which is only expressed in about one-fifth of GC patients [8].

CDH17, also known as liver intestine (LI)-cadherin, is a unique member of cadherin superfamily due to its long extracellular domain (seven cadherin domains) and a short cytoplasmic tail (20 aa residues), which is distinct from classical cadherins with five cadherin domains and a more than 100 aa cytoplasmic tail [9]. In physiological conditions, expression of CDH17 in human and mice is mainly restricted to the epithelial cells in small intestine and colon, but not vital organs such as liver, stomach, heart, lung and brain [10, 11]. Functionally, CDH17 is involved in intercellular adhesion to maintain tissue integrity and water absorption through regulating the intercellular cleft in a Ca^2+^-dependent manner [12]. Pathophysiologically, the expression of CDH17 has been extensively explored in various cancers from digestive system. Its expression is upregulated in gastric cancer (GC) [13, 14], colorectal cancer (CRC) [15], hepatocarcinoma (HCC) [16], pancreatic cancer (PC) [11], and neuroendocrine cancer [17]. Knockdown of CDH17 suppresses tumor development and metastasis in GC [14, 18], HCC [16], CRC [19] and PC [11]. Thus, CDH17 has been regarded as a cancer biomarker for prognosis and an oncogene for cancer intervention. Different CDH17 therapeutic formats were tested with favorable outcome in pre-clinical setting, including CDH17 monoclonal antibodies (mAb), CDH17 antibody conjugated with toxin saporin [20], TRAIL [15] or IRDye 700 [21], and CDH17 CAR T cells [15, 20, 22]. Most of these studies utilized the conventional full-length antibodies to target CDH17. The production of full-length antibodies based on mammalian cell expression systems is normally time and cost consuming, and the large molecular mass of full-length antibodies (~150KD) is an adverse factor which prevents the antibodies from the penetration into the tumor mass [23]. Furthermore, full-length antibodies conjugated with other protein drugs could further enlarge protein size, which might result in less accumulation of antibody conjugates into tumor tissues and thus impair the efficacy of therapy. Hence, it is necessary to develop smaller targeting proteins with comparable affinity to conventional antibodies to enhance the tumor tissue penetration and maximize the therapeutic effects.

Nanobody, mainly engineered from camelid heavy-chain only antibodies (hcAbs), is a relatively new type of small antibody which comprises single heavy chain variable domains (VHHs) without light chains and constant regions of conventional antibodies [24]. Despite of its smaller size (~ 15 kDa) accounting for ~ 1/10th of full-length IgG, nanobody retains high antigen binding affinity and specificity [25]. The tiny format endows nanobody with multiple unique advantages over conventional antibody, such as good tissue penetration power, rapid clearance, ease of production and modification, high stability and less immunogenicity [26, 27]. Given various advantages of nanobody over the conventional antibody, there are growing interest to develop the imaging and therapeutic modalities through nanobodies with diverse modification [23].

In the present study, we identified two CDH17 nanobodies and used them to develop imaging and therapeutic strategies for gastric cancer expressing CDH17. We demonstrated that CDH17 nanobody can be used for gastric cancer imaging. In addition, the nanobody can be fused with the potent toxin PE38, a truncated Pseudomonas exotoxin A, to produce native soluble recombinant protein drug which could effectively control the progression of gastric cancer in both CDX and PDX models. Our findings confirm the potential of CDH17 as a target for gastric cancer or other cancers overexpressing CDH17 by using nanobodies and encourage clinical translation of CDH17 nanobody-based targeted imaging and therapy.

## Materials and methods

### Cell lines and cell culture

Six cell lines (MKN45, AGS, TMK1, IM95, GES-1 and MDA-MB-231) were obtained from the American Type Culture Collection (Manassas, VA); Cell Bank of Chinese Academy of Sciences (Shanghai, China) and Fuheng Biology (Shanghai, China). MKN45, TMK1 and AGS cells were cultured in RPMI1640 (Gibco, USA) supplemented with 10% fetal bovine serum (FBS), 2 mM L-glutamine and 1% penicillin/streptomycin. IM95, GES-1, and MDA-MB-231 were maintained in high-glucose Dulbecco’s Modified Eagle’s Medium (DMEM) supplemented with 10% FBS, 2 mM L-glutamine, and 1% penicillin/streptomycin. All the cell lines were maintained at 37 °C in a humidified 5% CO_2_ incubator.

### Nanobody screening with an unimmunized naïve nanobody phage library

The nanobody screening was conducted as previously described [28]. Three rounds of biopanning were performed to obtain the antigen-specific VHH fragments with naïve nanobody phage library (Naïve VHH library) prepared with PMBC RNA from more than 100 alpacas (*Lama pacos*) (Shenzhen KangTi Life Technology Co., Ltd., China, KTSM-CND002). Briefly, the purified domain 1-3 of human CDH17 protein were coated on an immune tube at 40 μg/tube concentration overnight, followed by an incubation with 3% BSA-PBS solution for 1 h at RT. The VHH phage library was then incubated in the tube for 1 h at RT. Then, unbound phage clones were washed away with PBST (PBS + 0.1% Tween20). Trypsin (0.25 mg/ml) elution buffer was used to harvest the bound phages and was then neutralized with 4 mg/ml AEBSF. The eluted phage clones were subsequently amplified, rescued with M13 helper phages in *E. coli TG1* cells and precipitated with PEG–NaCl (20% PEG 800 and 2.5 M NaCl) and then resuspended in PBS. The phage library was collected and then used for titration and next-round screening. After three rounds of biopanning, 96 phage clones were randomly picked and amplified for phage ELISA.

Briefly, the microtiter plates were coated with 10 μg/ml purified CDH17 domain1-3, blocked with a 3% BSA solution, and then phage clones were added and the plates were incubated for 1 h at RT. The plates were washed three times with PBST (PBS + 0.1% Tween20) and finally incubated with an HRP-conjugated anti-M13 monoclonal antibody (Sino Biological, Beijing, China). Subsequently, the plates were washed again with PBST and then incubated with the TMB peroxidase substrate and reaction was stopped with 1 M HCl. The absorbance was then measured at 450 nm by an automated microplate reader (LabServ K3 TOUCH, Thermo Fisher Scientific, USA). The clones with more than 10-fold increase of absorbance value (CDH17 vs BSA) were regarded as positive. According to the ELISA data, 50 positive clones were finally identified and 36 sequences were obtained after sequencing.

### Protein purification

For the expression and purification of target protein, the recombinant plasmids pET-14B-CDH17-domain 1-3 (human) were transformed into BL21 (DE3) and then the bacterial clones were incubated at 37 °C and 225 rpm until reaching 0.6 of OD600 value, followed by induction with 0.2 mM IPTG at 16 °C and 225 rpm overnight. The cultures were pelleted with 8000 g for 15 min at 4 °C. Cell pellets were dissolved in lysis buffer (300 mM NaCl, 50 mM NaH_2_PO_4_, 10 mM imidazole, pH 8.0, 1 mM PMSF) and crushed at low temperature and high pressure for 3 times. The lysate was spun down for 45 min at 12,000×g, and the supernatants were loaded on a gravity column with 1 mL Ni-NTA agarose resin (Qiagen, Germany). The protein-bound resin was washed with 50 ml Wash Buffer I (300 mM NaCl, 50 mM NaH_2_PO_4_, 20 mM imidazole, pH 8.0, 1 mM PMSF) and 50 mL Wash Buffer II (300 mM NaCl, 50 mM NaH_2_PO_4_, 40 mM imidazole, pH 8.0, 1 mM PMSF) and then eluted with 25 ml Elution Buffer (300 mM NaCl, 50 mM NaH_2_PO_4_, 250 mM imidazole, pH 8.0, 1 mM PMSF). Finally, the eluate was fractionated by Superdex-150 gel with AKTA Pure System (GE Healthcare Life Sciences, USA) in 1 × PBS. The purified protein was identified by SDS-PAGE, and then quickly frozen in liquid nitrogen and restored in − 80 °C until use.

The nanobodies were purified by Ni-NTA agarose resin as described above for purification of CDH17 protein. The nanobodies sequences were inserted pColdII vector (Takara Bio) and a HA tag was integrated into nanobody sequence at C terminal with a GA linker. A cysteine was attached at the end of HA tag for further nanobody modification. The purified nanobody proteins were analyzed and identified by western-blot probed with 6 × His tag and HA tag antibodies.

The expression and purification of E8-PE38 or E8-PE38 mut fusion proteins were conducted with the similar procedures with nanobodies.

### Cell ELISA

To analyze the binding activity of A1 and E8 nanobodies to gastric cell lines, cell ELISA was performed as follows. In brief, gastric cell lines (TMK1, AGS, MKN45 and IM95) were cultured in 96-well plates at a cell density of 5 × 10^4^/well overnight. The cells were fixed with 4% paraformaldehyde for 5 min and followed by an incubation with 4% donkey serum solution for 1 h at RT. The nanobodies were diluted from 4000 nM to 62.5 nM with 1 × PBST and incubated with the plates at RT for 1 h. The plates were then washed three times with PBST (PBS + 0.1% Tween20) and a mouse anti-HA tag antibody (Creative Biomart, USA) was added for 1 h at RT. Afterwards, the plates were washed and incubated with a donkey anti-mouse IgG antibody conjugated with Alexa Fluro 488 (Invitrogen, USA) at RT for 1 h. The plates were washed with PBST for three times and the fluorescence intensity was measured under 488 nm laser channel by Sapphire Capture system (Sapphire, USA).

### Nanobody labeling with IR-800

The IR800 dye labeled nanobody was applied for in vivo imaging. Briefly, E8 or Control nanobody was diluted to 1 mg/mL in PBS (pH 6.5). IR800-mal were added and incubated for 2 h at room temperature in the dark. The unconjugated dye was removed using a 10 K molecular-weight cutoff (MWCO) spin desalting column. Concentrations of the nanobody were measured using a NanoDrop One spectrophotometer (Thermo Scientific, USA).

### Tumor imaging in vivo and ex vivo

When tumors reached approximately 500 mm^3^, the MKN45 tumor-bearing mice were randomly divided into two groups (*n* = 3) and injected intravenously 100 μg E8 Nb-IR800 and Con Nb-IR800 respectively. At different time intervals, the mice were subjected to fluorescence scanning using an IVIS Spectrum imaging system (PerkinElmer, USA). The mice were sacrificed at the end of the experiment, and major organs (heart, liver, spleen, lung, kidney) and tumors were harvested for ex vivo fluorescence scanning.

### In vitro cell viability assay

In order to verify the targeted toxicity of E8-PE38 on gastric cancer cells, CCK8 (Cell Counting Kit-8) assay was conducted to determine the cytotoxicity and half-maximal inhibitory concentration values (IC50) of different recombinant immunotoxin proteins. MKN45, TMK1, AGS, and IM95 cells were seeded into 96-well plates at 5 × 10^3^ cells in 100 μl culture media per well, respectively. After overnight culturing in a humidified incubator (at 37 °C, 5% CO_2_), cells were gently rinsed once with PBS; and then different concentrations (0, 1.95, 3.91, 7.8125, 15.625, 31.25, 62.5, 125, 250, 500 nM in 100 μl culture media) of the purified E8, E8-PE38, Con-PE38 or E8-PE38 mut were added to wells, respectively. The plates were incubated for 72 h at 37 °C in the humidified incubator. Then 10 μl CCK-8 solution (Abcam, China) per well was carefully added into the plates without the introduction of bubbles. Plates were incubated for another 1 h at 37 °C. Absorbance was measured at 450 nm by an automated microplate reader (LabServ K3 TOUCH, Thermo Fisher Scientific, USA) after shaking. Graphs for cell viability and IC50 values were analyzed with Graph prism.

### Mouse xenograft models and treatment

All experiments on animals in the present study were done following the approved protocol by Institutional Animal Care and Use Committee (IACUC) of the Shenzhen People’s hospital and were carried out in accordance with relevant institutional and national guidelines and regulations. Balb/C nude mice and NCG mice (NOD/ShiLtJGpt-*Prkdc*^*em26Cd52*^*Il2rg*^*em26Cd22*^/Gpt) at 8 weeks old were purchased from Gempharmatech (Guangzhou, China) and were maintained under pathogen-free conditions in the animal center of the Shenzhen People’s hospital. Mice were euthanized when showed obvious signs of discomfort or when maximal tumor size reached 2000 mm^3^.

Gastric cancer cells MKN45 (4 × 10^6^ cells) or TMK1 (5 × 10^6^ cells) were suspended in 100 μL PBS and injected subcutaneously into the right flank of mice. Tumor size was measured with vernier calipers and calculated using the following formula: (length × width^2^)/2.

In order to determine the appropriate dose of E8-PE38, MKN45 tumor-bearing mice were first selected for anti-tumor study. In brief, tumor-bearing mice were randomly divided into control group (PBS), low-dose group (0.4 mg/kg E8-PE38), and high-dose group (0.6 mg/kg E8-PE38) when the tumor size reached approximately 150 mm^3^ (*n* = 4-5 per group). Drugs were administered intravenously as indicated schedule in Fig. 4d every other day for seven injections. During treatment, tumor size and body weight in mice were monitored. For survival study, mice were sacrificed when tumor size reached 2000 mm^3^.

To confirm the therapeutic effect of E8-PE38, MKN45 tumor-bearing mice were randomly divided into 4 groups and treated with PBS, E8 (0.15 mg/kg), E8-PE38 Mut (0.6 mg/kg), and E8-PE38 (0.6 mg/kg) (*n* = 5-6 per group). The mice were treated as schedule above for seven injections. During treatment, tumor size and body weight were recorded. After treatment, the major organs including heart, liver, spleen, lung, kidney, and tumor were collected, and frozen sections were prepared and analyzed by H&E staining, Ki67 and TUNEL staining. In the TMK1 tumor model, tumor-bearing mice were randomly divided into 4 groups and treated with PBS, E8 (0.1 mg/kg), E8-PE38 Mut (0.4 mg/kg), and E8-PE38 (0.4 mg/kg) for tumor growth inhibition and survival analysis (n = 5 per group).

For combination therapy, 5-FU (25 mg/kg) and E8-PE38 (0.4 mg/kg) were respectively administered to tumor-bearing mice (MKN45) every other day for seven injections for each drug as scheduled in Fig. S7. Tumor growth and volume were monitored every 2 days until tumor size reached 2000 mm^3^.

### Patient derived gastric xenograft model and treatment

A piece of fresh PDX gastric cancer tissue (2nd passage) was kindly gifted by Dr. Yuanqiao He from Nanchang University and was confirmed the expression of CDH17 by IHC. The sample was derived from a patient with stage III C gastric adenocarcinoma. Informed consent was obtained from the patient, and the procedures involving human samples were approved by the medical ethical committee of the Shenzhen People’s Hospital and Nanchang University. Received PDX tissue was rapidly cut into 3x3x3 mm fragment in ice, and subsequently implanted subcutaneously in right forelimb of a NCG mouse to amplify the tumor cells. The tumor was exercised and cut into small pieces when grew to 800 ~ 1000 mm^3^, and subcutaneously inoculated into the right forelimb of new NCG mice. When those tumors reached ~150 mm^3^, mice were randomly divided into control group (PBS), low-dose group (0.4 mg/kg E8-PE38), and high-dose group (0.6 mg/kg E8-PE38) for anti-tumor study and survival assessment (*n* = 6-7 per group).

### Immunostaining

To analyze cell membrane expression of CDH17 or the co-localization of nanobodies with CDH17 on gastric cells, immunofluorescence of CDH17 and nanobody was conducted. In brief, gastric cell lines MKN45 and IM95 were cultured on a 24-well glass slide plate at a cell density of 1 × 10^5^/well overnight. The cells were fixed with 4% paraformaldehyde for 5 min and followed by an incubation with 4% donkey serum solution for 1 h at 37 °C. A1 and E8 nanobodies were diluted with 1 × PBST and incubated at RT for 1 h. The plates were then washed three times with PBST (PBS + 0.1% Tween20) and finally incubated with mouse anti-HA tag antibody (Creative Biomart, USA) and rabbit anti-CDH17 antibody at RT for 1 h. Next, the plates were washed again and then detected with Alexa Fluro 594-conjugated donkey anti-mouse IgG antibody (Invitrogen, USA) and Alexa Fluro 488-conjugated donkey anti-Rabbit IgG antibody (Invitrogen, USA) at RT for 1 h. Finally, the plates were washed again with PBST and cell nuclei were stained with 5 μg/ml DAPI. The Immunofluorescence of CDH17 and nanobody was analyzed by laser scanning confocal microscope (Leica TCS SP8, Germany).

For nanobody internalization analysis, MKN45 cancer cells were seeded in coverslips overnight and then were incubated with 4 μΜ nanobodies for 1 hour and 3 hours. Next surface bound nanobodies were removed with glycine buffer (0.2 M, pH 2.5). Cells were then fixed with 4% PFA for 15 mins and permeabilized with 0.1% triton X-100 for 5 mins. Nanobodies internalized into the cells were then detected with a HA-tag antibody and a secondary fluorescent antibody.

To analyze and evaluate the expression level of CDH17 in gastric cancer tissue, immunohistochemistry of CDH17 was performed in a gastric cancer tissue microarray. A gastric cancer tissue microarray consisting of 79 samples with clinical information was obtained from a biobank (OUTDO BIOTECH, Shanghai, China). The sections were stained with a rabbit polyclonal antibody against CDH17 (Abclonal, USA). CDH17 detection was performed by biotin-conjugated goat anti-rabbit IgG secondary antibody and ABC kit (Vector, USA) followed by colorimetric detection using diaminobenzidine (DAB; Vector, USA). The images were obtained using a 3DHISTECH™ scanner (Sysmex, UK). The pictures were analyzed as described previously [29]. The procedure involving human samples was approved by the medical ethical committee of the Shenzhen People’s Hospital. The clinical information is described in supplementary Table 1.

### Statistical analysis

All the data are present as mean ± SEM unless otherwise specified. GraphPad Prism software was used to conduct the statistical analysis for all the data. A two-tailed student’s t-test was utilized to analyze the difference between two samples. Tumor weight and various toxicological parameters among four groups were analyzed with one-way ANOVA. In vitro cell viability and tumor growth curves were evaluated with two-way ANOVA. Survival curves between groups were compared with a log-rank test. *P* < 0.05 was considered as significant and asterisks indicate the significant difference (*p < 0.05, **p < 0.01, ***p < 0.001, ****p < 0.0001).

## Results

### Recapitulation of CDH17 overexpression in gastric cancer and screening of CDH17 nanobodies

**Fig. 1:**
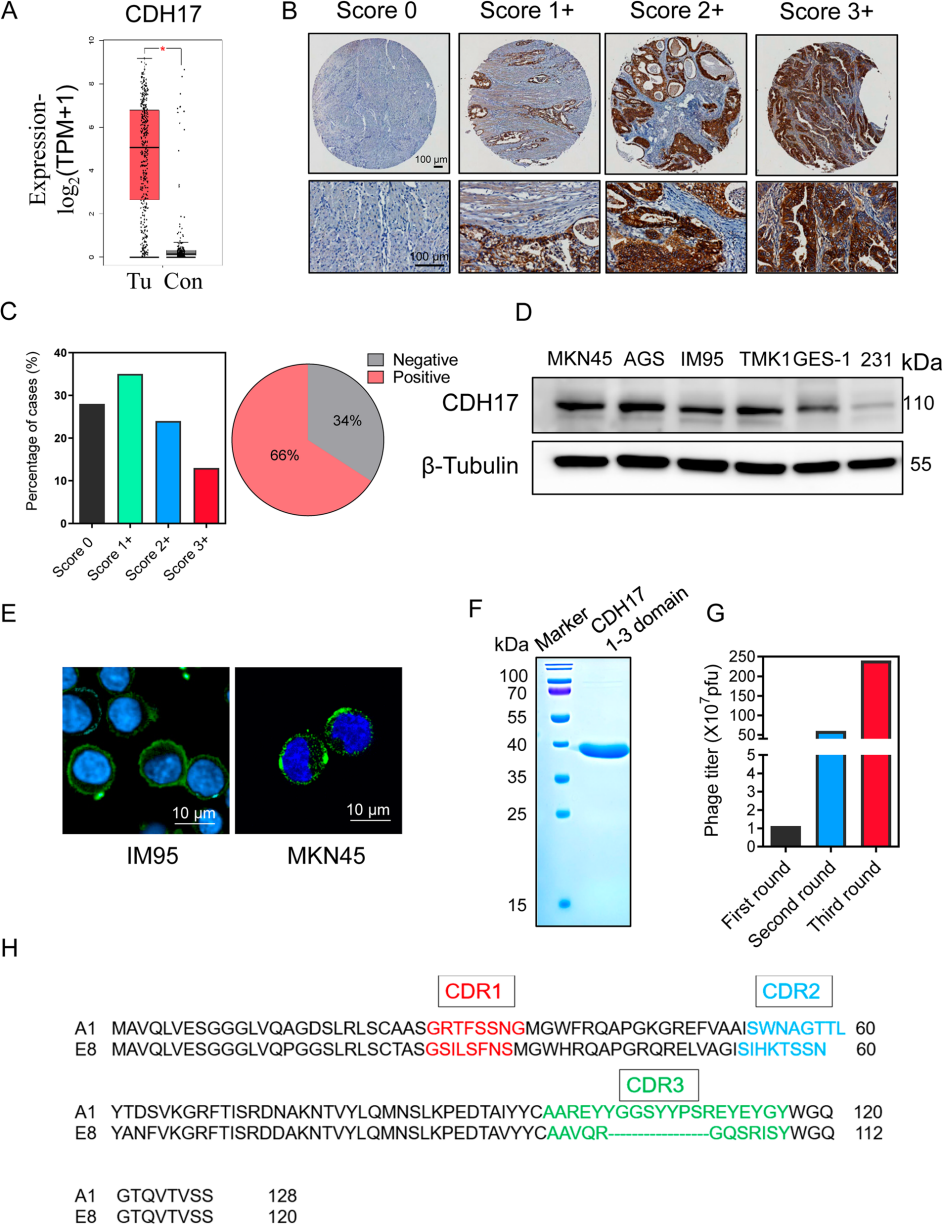
Recapitulation of CDH17 expression in gastric cancer samples and isolation of CDH17 nanobodies. **a** CDH17 RNA expression (TPM, RNAseq) in gastric cancers and normal stomach controls. *n* = 408 (tumors) and 211(controls). **b** CDH17 protein expression assessed by IHC in gastric TMA samples. *n* = 79. Scale bars, 100 μm. **c** Score percentage (left) and positivity rate (right) of CDH17 expression analyzed from **b**. **d** CDH17 protein expression in cell lines determined with western blot. **e** CDH17 immunostaining in cell membrane of IM95 and MKN45 cell lines. **f** SDS-PAGE gel analysis of recombinant CDH17 domain 1-3. **g** Nanobody screening against CDH17 domain 1-3 with phage display technology. **h** Sequences alignment of isolated A1 and E8 nanobodies with highlighted CDR regions

CDH17 expression has been documented in GC samples and used with other markers such as CDX2 and GPA33 for prognostic prediction in GC patients [13, 30]. To confirm that CDH17 is a better targeting molecule in gastric cancer, we first compared the mRNA expression of three markers (CDH17, HER2 and VEGFR2) in gastric cancer using Genotype Tissue Expression (GTEx) and the Cancer Genome Atlas (TCGA) databases. Although HER2 and VEGFR2 have been targeted for GC therapy, the mRNA data suggest that the discrepancy of CDH17 expression between GCs and normal stomachs is much larger than those of HER2 and VEGFR2, and normal gastric tissues have extremely low CDH17 mRNA (Fig. 1a, S1a). Next, we examined the expression of CDH17 protein in a tissue microarray (TMA) sample containing 79 GC cases with different pathological characteristics (Table S1). The expression levels of CDH17 ranged from score 0 (negative) to score 3 + (strong). Most of the cases (54.4%) showed moderate expression of CDH17 (score 1+ and 2+) mainly localized in cell membrane and cytoplasm (Fig. 1b). Approximately 11.4% GC cases displayed strong (score 3+) expression of CDH17(Fig. 1b and c). CDH17-positive gastric cancers account for approximately 66% in our tested cohort, which is consistent with previous reports (Fig. 1c) [30, 31]. CDH17 expression was further evaluated in four GC cell lines, one gastric normal epithelial cell line GES-1 and a negative control cell line MDA-MB-231. Compared with GES-1 and MDA-MB-231 cells (Fig. 1d), four gastric cancer cell lines showed significant expression of CDH17, and membrane staining of CDH17 can be clearly identified in GC cell lines (Fig. 1e). Our results combined with previous reports concretely reveal that CDH17 is a potential molecule suitable for targeted imaging and drug delivery in GC.

To screen the nanobodies targeting CDH17, we next cloned the extracellular domain 1-3 of CDH17 and purified the recombinant protein from *E. coli.* An approximate 40 kD soluble protein was obtained with high purity (Fig. 1f). The protein was then applied for nanobody screening through phage display technology. After three rounds of screening, the sub-library in third round was enriched more than 50 folds when compared with the sub-library in the first round, indicating that the potential binders were largely amplified and recovered (Fig. 1g). The preliminary phage ELISA identified more than 50 positive binders bound to CDH17 from 96 phage clones (Fig. S1b) and a total of 36 nanobody clones were successfully sequenced. Further analysis identified two highly enriched nanobody sequences, termed A1(32/36) and E8(4/36). These two nanobodies harbored three different complementary determined regions (CDRs), and E8 nanobody contained a shorter CDR3 fragment when compared with A1 nanobody (Fig. 1h, S1c).

### Characterization of CDH7 nanobodies

**Fig. 2:**
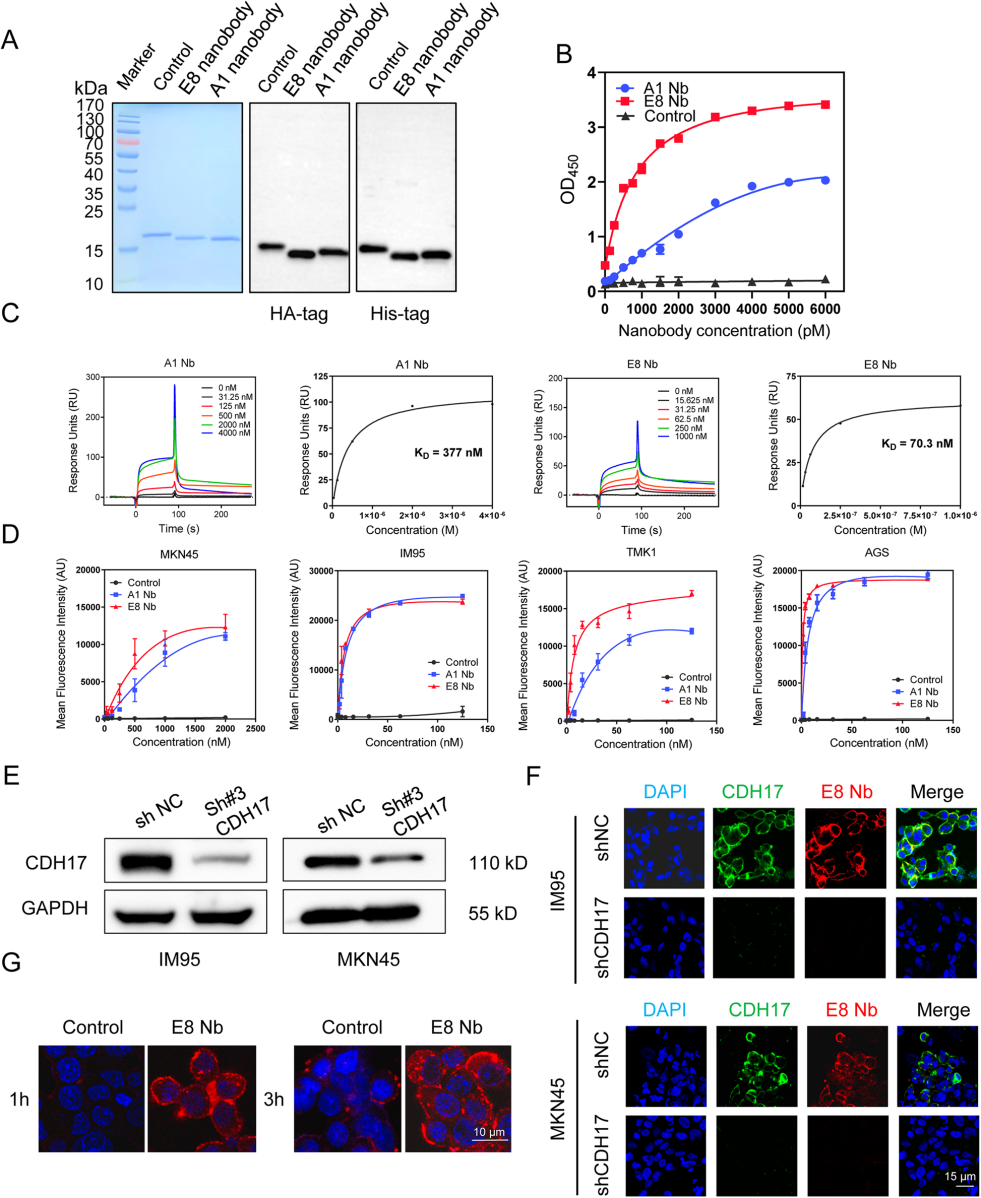
Characterization of A1 and E8 nanobodies against CDH17. **a** SAD-PAGE analysis of purified nanobodies (left) and nanobody confirmation with HA antibody (middle) and His antibody (right). The molecular weight for three nanobodies ranged from 15 kDa to 18 kDa. **b** ELISA analysis of binding ability of A1 and E8 nanobodies to CDH17 domain 1-3 (*n* = 2). Data are representative of two independent experiments. **c** Determination of binding affinity to CDH17 protein by SPR analysis. The equilibrium dissociation constant K_D_ was 377 nM (A1) and 70.3 nM (E8) respectively. **d** Binding activity of A1 and E8 nanobodies in CDH17-positve cells (MKN45, IM95, TMK1 and AGS) assessed by fluorescent cell ELISA (*n* = 3). Both of nanobodies could recognize CDH17 protein expressed in cell membrane, while E8 nanobody shows a better performance. Data are expressed as mean ± SEM. **e** Validation of knockdown CDH17 with shRNA#3 in IM95 and MKN45 cells determined with western blot. **f** Binding specificity of E8 nanobody to CDH17 in CDH17-overexpressing and -knockdown cell lines respectively. E8 nanobody cannot obviously stain the cells with knockdown CDH17, indicating the great specificity of E8 nanobody to CDH17. **g** Internalization analysis of E8 nanobody with one-hour and three-hour incubations followed with HA-tag staining. An irrelevant nanobody as a control was used for all the assays above

To verify the binding ability of A1 and E8 nanobodies to CDH17, we cloned these nanobody sequences and an irrelevant control nanobody sequence into a pCold vector, and simultaneously incorporated the HA tag at the C terminal for subsequent detection and a cysteine amino acid at the end of HA tag for further nanobody modification. The three soluble nanobody proteins were purified using *E. coli* expression system and was further confirmed with HA tag antibody and His tag antibody. The molecular mass of these three nanobodies ranged from 15 to 18 kDa (Fig. 2a).

Subsequently, the binding ability to CDH17 was confirmed for A1 and E8 nanobodies with ELISA. Both of nanobodies could bind to CDH17 domain 1-3 and E8 nanobody exhibited a better binding curve while the control nanobody did not show any binding activity (Fig. 2b). The binding affinity was then measured with surface plasmon resonance (SPR). Consistent with ELISA results, E8 nanobody displayed a stronger binding affinity with K_D_ value of 70.3 nM compared with 377 nM of A1 nanobody (Fig. 2c).

To assess whether A1 and E8 nanobodies can recognize CDH17 expressed in GC cell lines, both nanobodies as well as a control nanobody were evaluated by cell ELISA assay. Both of A1 and E8 nanobodies showed strong fluorescent signal in a dose-dependent manner in all four GC cell lines but not the control nanobody (Fig. 2d, S2a), indicating that both of nanobodies can recognize the natural CDH17 localized in the membrane of GC cell lines. To determine the binding specificity, shRNAs were used to knockdown CDH17 expression in MKN45 and IM95 cell lines. All three shRNA sequences targeting different regions significantly knocked down the expression of CDH17 and shRNA#3 indicated the best efficiency (Fig. S2c, Fig. 2e and Table S2). Cell ELISA data revealed that A1 and E8 nanobodies showed significantly reduced binding activity to CDH17-knockdown GC cell lines with three shRNA sequences and binding signal was almost undetectable in MKN45 cells knocked down CDH17 with shRNA#3 (Fig. S2b), thereby demonstrating that A1 and E8 nanobody could specifically recognize CDH17 protein. Based on overall binding affinity determined by protein ELISA, SPR and cell ELISA, E8 nanobody exhibited a better binding activity than A1 nanobody, and was further investigated for the following studies. E8 nanobody was further confirmed the co-localization with CDH17 in the cell membrane of two GC cell lines MKN45 and IM95, and knockdown CDH17 completely eliminated the binding signal of E8 nanobody in both of cell lines (Fig. 2f), indicating that E8 nanobody could bind to CDH17 with a great specificity and affinity. Internalization is an essential determinant for nanobody to efficiently deliver drugs into cancer cells. We thus tested the internalization of E8 nanobody in MKN45 cells. E8 nanobody could be internalized in one-hour incubation and the internalization was significantly enhanced in three-hour incubation in MKN45 cells (Fig. 2g). Taken together, our results demonstrate that A1 and E8 nanobodies can efficiently bind to CDH17, and specifically recognize the CDH17-overexpressing GC cell lines, and internalization of E8 nanobody endows it with potential applications for GC imaging and targeted therapy.

### E8 nanobody can image gastric cancer ex vivo and in vivo

**Fig. 3:**
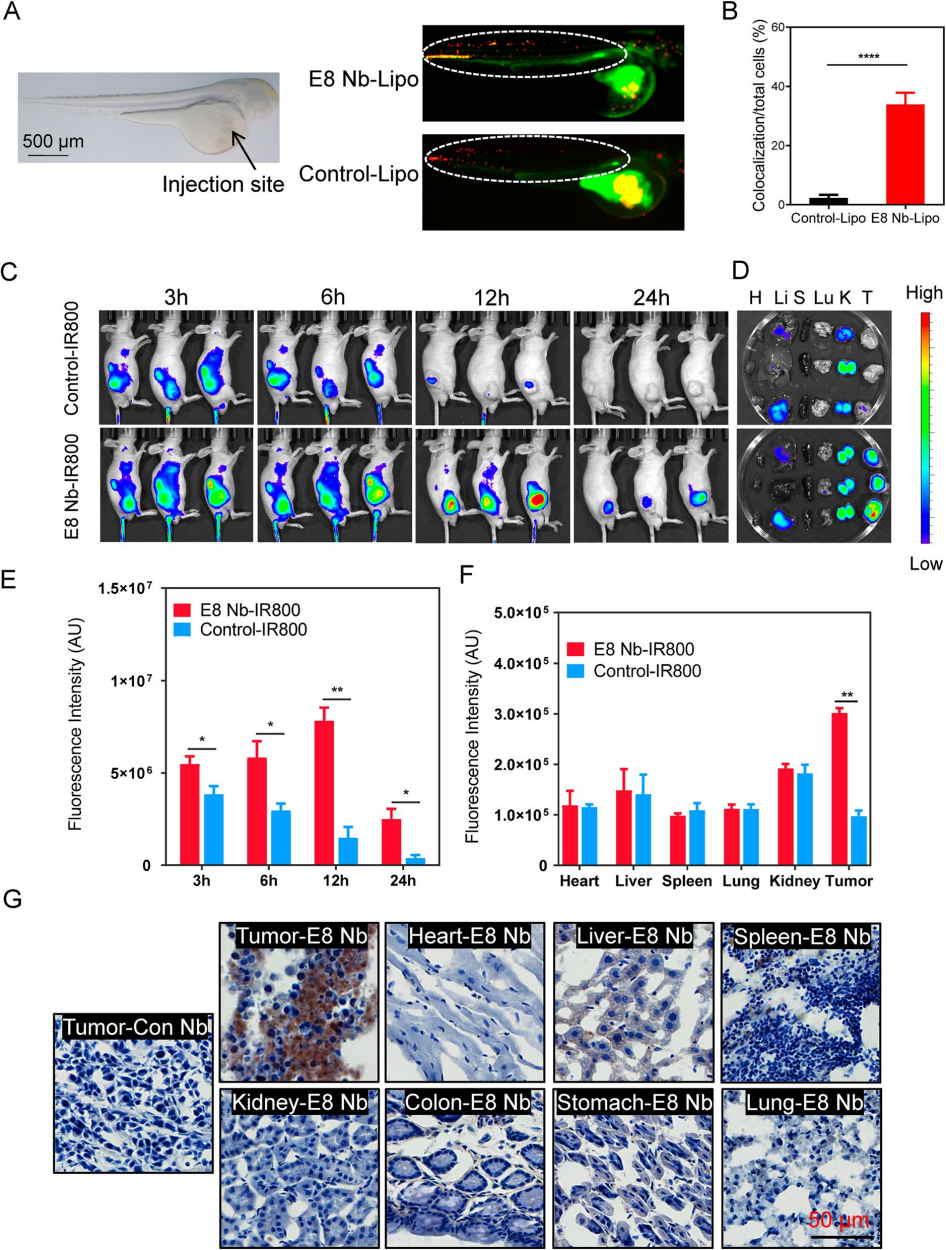
Gastric cancer imaging ex vivo and in vivo by CDH17 nanobody E8. **a** E8 nanobody (green) co-localization with CDH17-positve MKN45 cells (red) in zebrafish embryos. The appearance of zebrafish embryos (left) and co-localization of nanobody with cells (right). Dashed circles indicated the areas for quantification. **b** Quantification of co-localization (yellow) ratio to total red cells (*n* = 10, ****p < 0.0001, two-tailed student’s t test). Data are present as mean ± SEM. **c** and **e** Imaging of in vivo tumor-bearing mice with IR-800-labelled nanobodies in different time points (**c**, *n* = 3). Quantification analysis indicated that E8 nanobody in tumors produced significantly stronger signals as compared with control nanobody at each time point (**e**, *n* = 3, *p < 0.05, **p < 0.01, two-tailed student’s t-test). **d** and **f** Ex vivo imaging of major organs dissected from in vivo imaged mice in **c** (**d**, *n* = 3). Imaging quantification disclosed the strongest signals in E8-treated tumor tissues than all the control organs from both groups (**f**, *n* = 3, **p < 0.01, two-tailed student’s t-test). **g** Nanobody tissue distribution 12 hours after intravenous administration. Scale bars:50 μm. E8 nanobody could specifically accumulate into CDH17-positive tumor mass. Liver tissues showed some weak staining due to unspecific phagocytosis

We subsequently evaluated the ability of E8 nanobody for tumor imaging in zebrafish embryos and mouse xenograft models. MKN45 cells were labeled with CM-DiI dye and nanobodies were conjugated with liposomes encapsulated FITC. The mixture of MKN45 cells with nanobody-liposomes (Nb-lipo) was injected into zebrafish embryos through common cardinal veins (Fig. 3a). Zebrafish embryos were observed under microscopy and images were captured 30 min postinjection. Zebrafish embryos injected with E8 Nb-lipo showed more co-localization yellow dots than those in zebrafish embryos injected with control Nb-lipo (Fig. 3a and b), indicating that more E8 nanobody molecules bound to CDH17 expressed on MNK45 cell membrane.

Given the strong binding signal in zebrafish embryo model for E8 nanobody, we further assessed in vivo imaging ability of E8 nanobody. IRDye800cw dye (IR800) was employed to label the nanobodies through the reaction of cysteine at the end of C terminal of nanobodies with maleimide of IR800. The resultant products can be detected in SDS-PAGE gel under 780 nm excitation (Fig. S3a). Nanobody-IR800 was systemically injected into the tumor-bearing mice and then near infrared (NIR) fluorescent images were captured using IVIS imaging system under different time points post-injection. As shown in Fig. 3c, the fluorescent signals were gradually increased in tumor tissues treated with E8-IR800 from 3 h to 12 h, and then were declined 24 h post-injection (Fig. 3e). Compared with control Nb-IR800, E8-IR800 produced much stronger fluorescent signals in tumor site at each time point (3 h, 6 h, 12 h and 24 h) post-injection, suggesting the superior specificity and binding activity of E8 nanobody in CDH17-overexpressing tumors. Control Nb-IR800 could also generate weak but detectable signals 3 h post-injection due to EPR (enhanced permeability and retention) effect, and then rapidly decayed in fluorescent intensity and completely disappeared 24 h post-injection (Fig. 3c and e). Ex vivo imaging from exercised organs 24 h post-injection revealed that tumor tissues treated with E8-IR800 indicated the strongest signals compared with control organs or tumors treated with control Nb-IR800 (Fig. 3d and f). Liver and kidney tissues from both of treatment groups displayed some fluorescent signals, implying that the nanobody-IR800 might be cleared from liver and kidneys.

Due to the strongest signal for E8-IR800 12 h postinjection, we next assessed the distribution and specificity of E8 antibody in various organs from tumor-bearing mice treated with E8 or control nanobody for 12 h. Nanobodies were injected into MKN45-induced tumor model and were allowed to circulate for 12 h. Various organs were collected after perfusion with PBS (20 ml) to remove non-specific nanobody accumulation, and nanobodies were stained with VHH antibody plus HA tag antibody for maximal signal amplification. The data revealed that E8 nanobody can be detected in tumor tissues, and the staining on important organs such as brain, heart, lung and stomach received E8 nanobody injection did not find visible positive staining, with the exception of liver tissues showing weak staining due to the unspecific phagocytosis of reticuloendothelial system (Fig. 3g); surprisedly, kidneys were not also shown strong staining signal although they are the main organs for nanobody clearance [32], which might result from the PBS perfusion before organ collection for non-specific nanobody removal. Tumors treated with control nanobody did not show positive staining as well. Further fluorescent staining in tumor tissues showed that E8 nanobody can be detected while no visible signal was found in control nanobody-injected tumors (Fig. S3b). Negative control without primary antibodies did not reveal any staining in tumor tissues (Fig. S3b). The results highlight the superb specificity and penetration ability of E8 nanobody in CDH17-overexpressing GC model.

Collectively, the present results disclose that E8 nanobody could target CDH17-overexpressing tumor tissues with excellent specificity and precision, and is a promising candidate agent for gastric cancer targeted imaging.

### E8/PE38 immunotoxin efficiently suppresses gastric cancers in vitro and in vivo

**Fig. 4:**
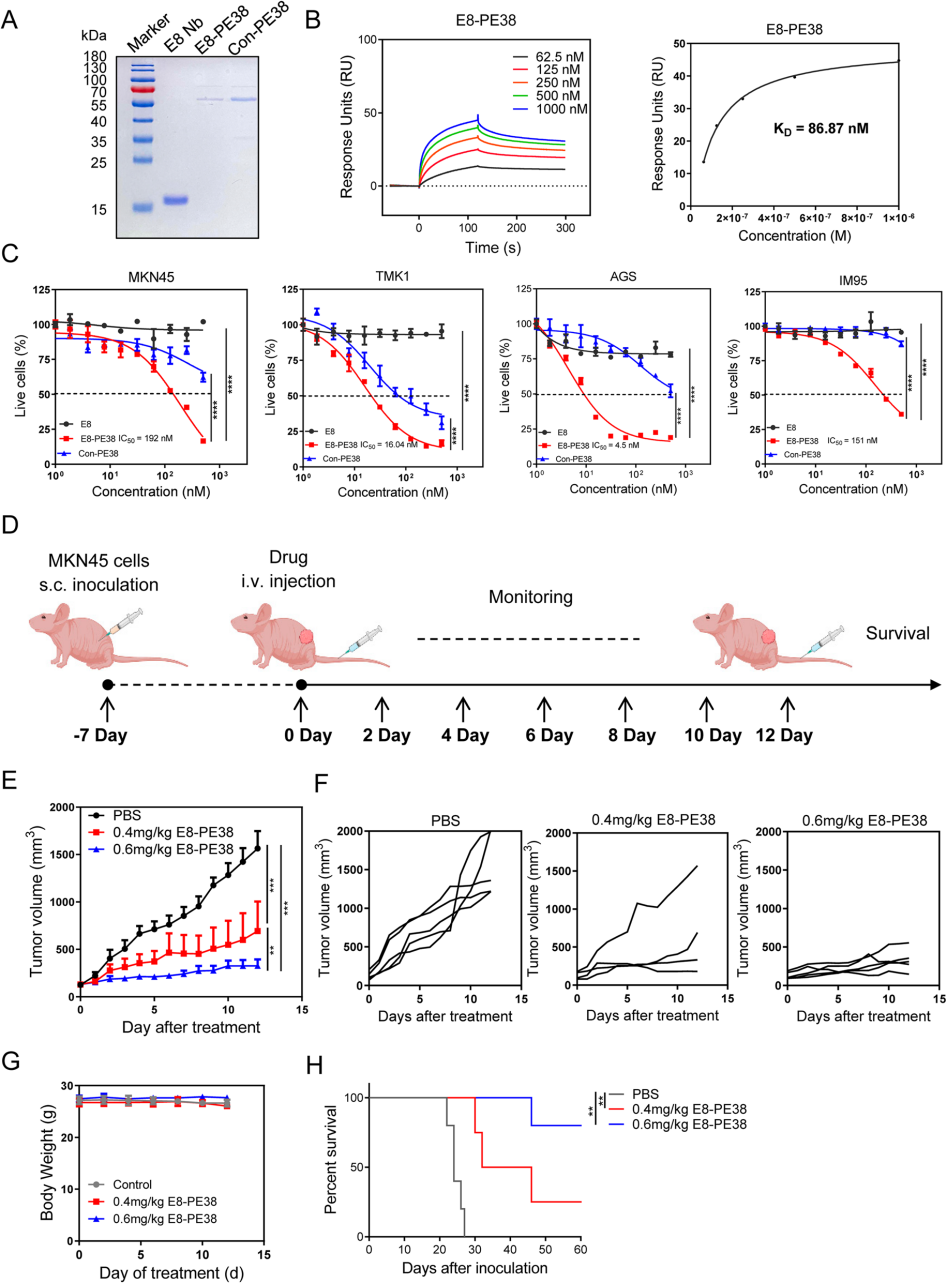
Activity evaluation of E8-PE38 immunotoxin in vitro and in vivo. **a** SDS-PAGE analysis of purified E8 nanobody (16 kDa), E8-PE38 (60 kDa) and Con-PE38 (60 kDa). **b** SPR analysis of E8-PE38 binding to CDH17. The K_D_ was 86.87 nM. **c** Cell viability detection after treatment with E8 nanobody alone, E8-PE38 and Con-PE38 in MKN45, TMK1, AGS and IM95 cells (*n* = 3, ****p < 0.0001, two-way ANOVA). Dashed line indicated the IC50 for E8-PE38 immunotoxin. **d** Schema of animal treatment schedule. **e** MKN45 tumor growth curves with the treatment of PBS, 0.4 or 0.6 mg/kg E8-PE38 (*n* = 4-5 per group, **P < 0.01, ***P < 0.001, two-way ANOVA). Mice were euthanized when tumor size reached 2000 mm^3^. **f** Individual tumor growth curves for three groups in **e**. **g** Body weight during the treatment from three groups in **e**. **h** Survival curves for treated mice in e (**p < 0.01, Log-rank (Mantel-Cox) test)

Having verified that E8 nanobody holds the great targeting ability against CDH17-overexpressing tumor for imaging, we next evaluated whether E8 nanobody could be applied as a drug delivery vehicle to treat gastric cancers. Truncated toxin PE38 was fused with E8 or control nanobodies, and the recombinant proteins were generated and purified as soluble native proteins with the approximate 60 kDa (Fig. 4a). SPR assay confirmed the binding ability of E8-PE38 immunotoxin to CDH17 with comparable K_D_ value (86.87 nM) to naked E8 nanobody (70.3 nM) (Fig. 4b). The cytotoxic effect of immunotoxins was examined by cell viability assay in four GC cell lines with different levels of CDH17 (see Fig. 1d). E8-PE38 immunotoxin showed potent cytotoxic activity and significantly reduced the cell proliferation in all four GC cell lines when compared with control Nb-PE38 toxin (Fig. 4c). E8 nanobody alone did not inhibit the cell growth (Fig. 4c). Those data implicated that E8 nanobody could deliver the toxin PE38 into CDH17-positive GC cells and enhance the cytotoxicity of anti-cancer payloads.

Based on the excellent cytotoxic activity in vitro, MKN45-induce subcutaneous tumor model was used to assess the anti-tumor efficacy of E8-PE38 immunotoxin. Due to the lethal effect of non-targeted PE38 to mice, the control Nb-PE38 or non-targeted PE38 was not used as a control drug. Here we first selected two doses of E8-PE38 to determine the anti-tumor effect of the immunotoxin as illustrated schedule in Fig. 4d. Both of dosages (0.4 mg kg^− 1^ and 0.6 mg kg^− 1^) could remarkably suppress tumor growth when compared with vehicle control, and higher dose of E8-PE38 (0.6 mg kg^− 1^) showed more homogenous inhibition for tumor growth (Fig. 4e and f). Meanwhile, there is no significant change in the body weight among the three groups throughout the treatment (Fig. 4g). Survival analysis also indicated that both of dosages significantly extended the survival of tumor-bearing mice as compared with PBS control (Fig. 4h). Together, these results demonstrate that E8 nanobody could efficiently deliver anti-cancer payloads into CDH17-expressing GC.

### E8-PE38 immunotoxin but not E8 nanobody produces the tumor inhibitory effect

**Fig. 5:**
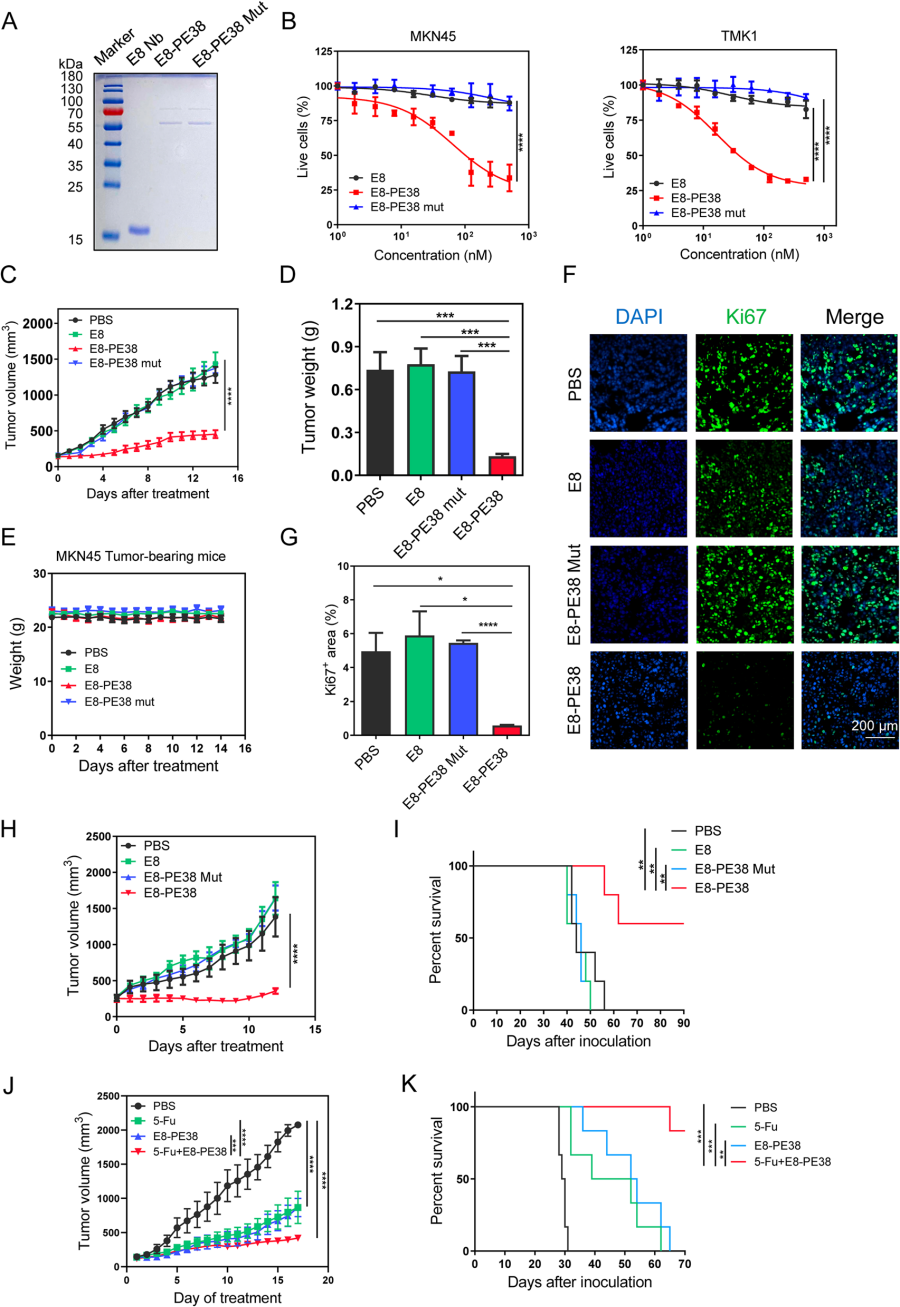
Toxin is the determinant causing tumor inhibition by E8-PE38. **a** SDS-PAGE analysis of purified E8 (16 kDa), E8-PE38 (60 kDa) and E8-PE38 mut (60 kDa). **b** Cell viability assay for MKN45 and TMK1 cells treated with E8, E8-PE38 and E8-PE38 mut (*n* = 3, ****p < 0.0001 compared with E8 or E8-PE38 mut, two-way ANNOVA). E8 nanobody alone and E8-PE38 mut did not show cytotoxic effect on cell proliferation. **c** MKN45 tumor growth curves from mice treated with PBS, E8-PE38 (0.6 mg/kg), E8-PE38 mut (0.6 mg/kg) or equal molar E8 nanobody (*n* = 5-6, ****p < 0.001 as compared with all three control groups, two-way ANOVA). E8 alone or E8-PE38 mut did not have any tumor inhibitory effect. E8-PE38 significantly suppressed tumor growth. **d** Tumor weight from treated mice at the end of treatment in **b** (*n* = 5-6, ***p < 0.001, one-way ANOVA). **e** Body weight during the treatment from four groups in **c**. **f** and **g** Ki67 immunostaining in tumor tissues collected from **c**. Tumor tissues treated with E8-PE38 markedly inhibited the Ki67 expression and no obvious change was detection in three control groups (n = 5-6, *p < 0.05, ***p < 0.001, one-way ANOVA). Scale bars: 200 μm. **h** Tumor growth curves from TMK1 tumors treated with PBS, E8-PE38 (0.4 mg/kg), E8-PE38 mut (0.4 mg/kg) or equal molar E8 nanobody (*n* = 5, ****p < 0.0001 as compared with all three control groups, two-way ANOVA). **i** Survival curves of treated mice from **h** (n = 5, **p < 0.01, Log-rank (Mantel-Cox) test). **j** Tumor growth curves from MNK45 tumor bearing mice received the treatment with PBS, 5-FU (25 mg/kg), E8-PE38 (0.4 mg/kg), and combination therapy (5-FU + E8-PE38) (*n* = 6, ***p < 0.001, ****p < 0.0001, two-way ANOVA). **k** Survival curves of tumor-bearing mice treated in **j** (*n*=6, **p<0.01, ***p<0.001, Log-rank (Mantel-Cox) test)

Knockdown CDH17 was reported to repress the growth of liver cancer and gastric cancer [14, 16]. Antibody against CDH17 plus cisplatin could control liver cancer progression [16, 33]. To exclude the effect of E8 nanobody in our study, we constructed a mutant PE38 (E553D) fused with E8 nanobody (E8-PE38 mut) to inactivate the PE38 activity [34]. Soluble E8-PE38 mut was purified from E.coli system with a band of 60 kDa (Fig. 5a). The in vitro cytotoxic activity was conducted with CCK-8 proliferation assay. E38-PE38 mut completely lost its activity against.

CDH17-positive cells MKN45 and TMK1(Fig. 5b). Consistent with previous data, E8-PE38 was highly toxic to both cell lines and E8 nanobody alone did not show any toxicity (Fig. 5b). We next determined the in vivo anti-tumor effect of E8 nanobody, E8-PE38 and E8-PE38 mut in two GC models implanted with MKN45 cells and TMK1 cells. For MKN45 model, mice were treated with 0.6 mg kg^− 1^ E8-PE38, E8-PE38 mut and equal molar concentration of E8 nanobody alone. Similarly, E8-PE38 immunotoxin significantly and homogenously inhibited the MKN45 tumor growth (Fig. 5c and S5A). However, E8-PE38 mut was entirely deprived of the inhibitory effect on the tumor growth and did not show any statistical difference from E8 nanobody alone and vehicle (Fig. 5c). Tumor weight at the end point of experiment also showed similar results that E8 nanobody alone and E8-PE38 mut did not harbor anti-tumor activity, but E8-PE38 significantly reduced the tumor weight (Fig. 5d). Meanwhile, 0.6 mg kg^− 1^ E8-PE38 did not affect the body weight of mice when compared with other three groups (Fig. 5e), indicating its good biosafety in tumor-bearing mice. To confirm the inhibitory effect of E8-PE38 in MKN45 tumors, cell proliferation and apoptosis were further assessed by Ki67 immunohistological staining and TUNEL assay. As shown in Fig. 5f and g, E8-PE38 immunotoxin resulted in significant reduction of Ki67 expression in tumor tissues when compared with other three groups. There was no obvious difference among vehicle, E8 nanobody alone and E8-PE38 mut in terms of Ki67 expression. TUNEL assay for cell apoptosis also confirmed that E8-PE38 immunotoxin induced massive cancer cell apoptosis but no obvious apoptosis was detected in control groups (Fig. S4a). These results indicate that E8-PE38 immunotoxin can potently repress gastric cancer growth through suppression of cell proliferation and induction of cell apoptosis. Systemic toxicological studies were conducted to determine the biosafety of E8-PE38 immunotoxin at the end of treatment. In addition to no significant change of body weight (Fig. 5e), blood cell testing and serum biochemistry analysis did not disclose any statistical difference in E8-PE38-treated mice compared with the three control groups (Fig. S6a and 5b). Histological analysis for major organs did not identify obvious morphology alteration among all the groups (Fig. S4b). These results demonstrate that E8-PE38 immunotoxin is a highly efficient drug to suppress CDH17-positive gastric cancer without detectable side effects in vivo.

In line with MKN45 tumor model, we achieved similar results for E8-PE38 immunotoxin in another GC model induced by TMK1 in NCG mice. Briefly, due to the better sensitivity of TMK1 to E8-PE38 (IC50, 16.04 nM) than MKN45 (192 nM), 0.4 mg kg^− 1^ E8-PE38 immunotoxin was tested using the same administration schedule as MKN45 tumor model (Fig. 4d). Tumor growth was almost retarded in mice treated with E8-PE38 immunotoxin as compared with controls vehicle, E8 nanobody alone or E8-PE38 mut (Fig. 5h and S5b). Tumor growth were comparable in the three control groups (Fig. 5h and S5b). E8-PE38 immunotoxin also markedly extended the survival time of mice, and there was no difference in survival time for vehicle, E8 nanobody alone and E8-PE38 mut (Fig. 5i). During the entire treatment, the body weight for all groups were not obviously impacted (Fig. S5c).

In addition, a combinational regimen of 5-FU and E8-PE38 were also evaluated in MKN45 tumor model with administration schedule shown in Fig.S7a. 5-FU is the first line clinical chemo drug for advanced GC. Moderate dosage for both drugs were tested to examine the additive anti-tumor efficacy. Moderate dose of 5-FU (25 mg/kg) and E8-PE38 (0.4 mg/kg) alone showed quite similar anti-tumor effect, whereas the combination therapy suggested maximal tumor growth inhibition and prolonged survival time (Fig. 5j, k and Fig. S7b). The results indicate that E8-PE38 could be applied on CDH17-positive GC cancers with 5-FU together, implying the great promise of E8-PE38 immunotoxin for GC therapy.

Taken together, our results demonstrates that E8 nanobody has the superb targeting ability towards CDH17-positive tumors and could efficiently deliver toxin PE38 to control cancer growth without detectable adverse events. We also verify that E8 nanobody is safe and non-toxic for in vivo administration in mice. The combination therapy of 5-FU and E8-PE38 might be a promising regimen for CDH17-positive GC.

### E8/PE38 immunotoxin inhibits gastric tumor from a PDX model

**Fig. 6:**
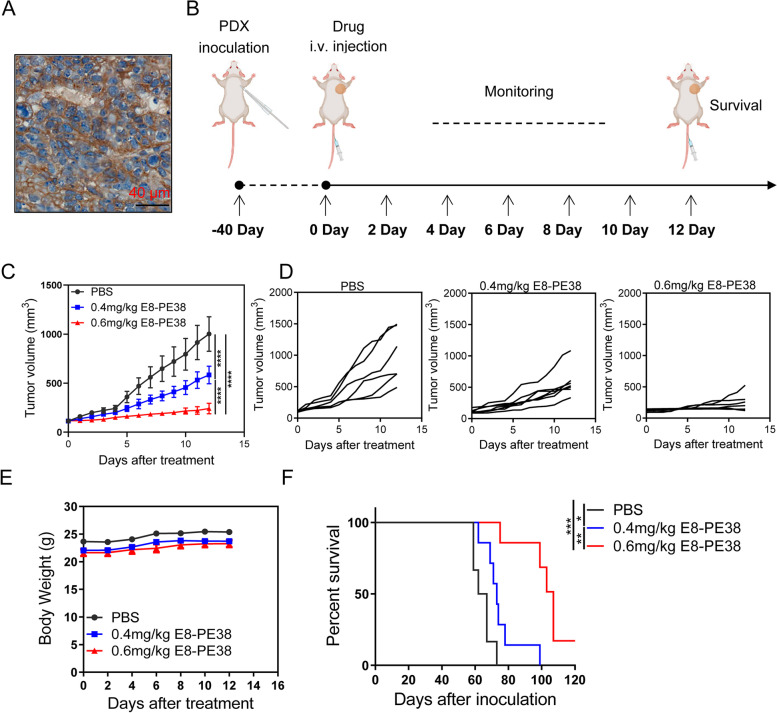
Anti-tumor effect of E8-PE38 in a PDX model. **a** CDH17 expression in a gastric PDX tumor. Scale bars: 40 μm. **b** Schema of treatment schedule in the PDX model. **c** Tumor growth curves from the treated PDX model with PBS, 0.4 or 0.6 mg/kg E8-PE38 immunotoxin (*n* = 6-7, ****p < 0.0001, two-way ANOVA). Both of dosages significantly inhibited the tumor growth. Higher dose of immunotoxin showed better anti-tumor efficacy. **d** Individual tumor growth curves for all three groups in **c**. **e** Body weight of the treated mice in **c**. **f** Survival curves of PDX mice treated in **c** (*n* = 6-7, *p < 0.05, **p < 0.01, ***p < 0.001, Log-rank (Mantel-Cox) test)

Patient-derived tumor xenografts (PDXs) are becoming the gold standard for clinical drug development due to the similarity of PDXs to original tumors dissected from patients [35]. To test whether E8-PE38 immunotoxin could produce similar influence on CDH7-positive PDX model, NCG mice were used to implant gastric cancer tissues dissected from a 3rd-passage PDX model which have been confirmed CDH17 expression (Fig. 6a). The PDX model was i.v. given E8-PE38 of 0.4 mg kg^− 1^ and 0.6 mg kg^− 1^ as illustrated schedule in Fig. 6b. Both dosages significantly repressed the PDX tumor growth and higher dose of E8-PE38 showed better anti-tumor effect with homogenous inhibition of the tumor growth (Fig. 6c and d). Body weight remained similar for all mice during the treatment (Fig. 6e). Two dosages of E8-PE38 could statistically increase the survival time, and it seemed that 0.6 mg kg^− 1^ E8-PE38 exhibited much better anti-tumor effect based on tumor burden and survival analysis (Fig. 6f). Conclusively, the superior tumor inhibitory effect of E8-PE8 immunotoxin on CDH17-positive PDX model supports that E8-PE38 immunotoxin holds great potential for further clinical development, and its high effectiveness and low systemic toxicity also prove that CDH17 is a targetable molecule for gastric cancer imaging and therapy.

## Discussion

Aberrant expression of CDH17 has been reported in a number of cancers including GC [13, 30], CRC [15, 36], esophageal cancer [37], hepatocellular carcinoma (HCC) [38], pancreatic cancer (PC) [37] and neuroendocrine tumor (NET) [17]. It was reported that approximate 70% gastric cancer and nearly all colorectal cancer express CDH17 with different levels [13, 15]. Consistent with published studies, TMA data in the present study showed that 66% of gastric cancer samples express CDH17. The aberrant expression has resulted in the attempts to use CDH17 as a target for cancer imaging and therapy [22, 39, 40]. In the present study, we identified two nanobodies specifically bound to CDH17 and E8 nanobody was extensively explored for gastric cancer imaging and targeted therapy due to its relatively higher affinity (~ 70 nM). Our findings unraveled that E8 nanobody could efficiently target tumor CDH17 molecule and produce specific fluorescence imaging signals within a short time in the tumor. It can also potently deliver the cytotoxic toxin into CDH17-positive cancer cells to suppress tumor growth. Our data demonstrate that CDH17 is a targetable protein for gastric cancer concerning imaging or therapy; and nanobody is a good strategy to design modalities against CDH17 protein.

Nanobody, as a relative new antibody format, is attracting growing interest in disease diagnosis and therapy due to its tiny size, ease of production and manipulation over conventional antibody. In the past 2 years, a diversity of nanobodies have been identified and applied for detection or neuralization of SARS-CoV-2 [41, 42], which has accelerated the development of the nanobody field. Nanobodies conjugated with various imaging contrasts have been attempted in the preclinical and clinical settings [43–45]. Fast tissue targeting ability, excellent tissue penetration and rapid clearance make nanobodies suitable for same-day imaging which is difficult for conventional antibodies due to large size and long half-life [25, 44, 46]. Nanobodies conjugated with isotopes have been applied for tumor imaging as a non-invasive diagnostic modality through targeting various tumor biomarkers such as HER2, Claudin18.2, fibronectin, etc. [28, 45, 47]. Normally, specific tumor imaging information could be visualized for nanobody-isotope conjugates within 6 hours and rapidly cleared by kidneys within 24 hours, which is significantly faster and more convenient compared with conventional antibodies whose conjugates need to wait for 4 to 6 days to obtain scanning window and requires longer time for complete clearance [45, 48]. On the other hand, although radio-isotopes are sensitive imaging probes applied for PET (Positron emission tomography) or SPECT (Single-photon emission computerized tomography) modality as a non-invasive imaging tool for disease diagnosis, radiation exposure is inevitable for both patients and physicians. Near infrared (NIR) fluorophores are becoming alternative options for tumor imaging due to deep tissue penetration, low cost, flexibility, non-radioactivity, and high target-to-background signals [25, 49]. Nanobodies modified with NIR dyes such as IRDye800cw through maleimide-cysteine site specific conjugation technology have been demonstrated their efficiency, stability and reproduction for in vivo imaging and imaging guided surgery [26, 43, 50, 51].

Recently, Fujiwara K et al. developed a full-length antibody D2101 conjugated with ^111^In isotope against CDH17 for gastric cancer imaging in a xenograft model [39]. It demonstrated that this conjugate took 96 hours to reach best high-contrast imaging, which cannot meet the same-day imaging requirement. Later, the same group reformatted the primary antibody into single chain Fv (scFv) with ~ 30 kDa to speed up the clearance and shorten the waiting time for imaging [40]. Unfortunately, even if the smaller format was developed, it still needed 24 hours to obtain highest tumor signal. In the present study, we labeled CDH17 nanobody E8 with IR-800 through site specific conjugation and tested the conjugate for imaging of CDH17-positive tumor model. The tumors showed clear imaging signals 3 hours post-injection and reached the peak at 12 hours post-injection. The signals have faded at 24 hours. These characteristics make E8 nanobody suitable for same-day imaging, and the imaging performance is much better than reported full-length antibody or smaller scFv against CDH17 [39, 40]. Kidneys should be the major organ for the clearance of E8 nanobody-IR800 since the most of conjugates are present in kidneys at 24 hours post-injection in addition to highest tumor accumulation (Fig. 3e). Therefore, our findings demonstrate that nanobody conjugates against CDH17 hold great potential for gastric cancer imaging and should be considered preferentially in lieu of conventional antibody for visualization of CDH17-postive gastric cancer. Further investigations should be explored whether E8 nanobody is able to be employed for imaging-guided gastric cancer surgery or for visualization of lymph node metastasis of CDH17-postive gastric cancer since lymph node metastasis is common in gastric cancer and CDH17 expression could be preserved in metastatic sites from primary tumors [15].

Immunotoxin consisting of an antibody moiety targeting cancer cell antigens and a toxin moiety for cell killing has been shown prominent anti-tumor effect, especially in blood cancers [52]. Lumoxiti comprising of a CD22-targeting scFv domain and a truncated Pseudomonas exotoxin A domain has been approved by FDA for relapsed or refractory hairy cell leukemia [53]. A serial of immunotoxins are being evaluated in the clinical trials for various cancers including solid tumors [53, 54]. The scFv format as a relatively smaller antibody fragment is commonly utilized as the targeting domain in the immunotoxin design. However, the immunotoxins normally need to be purified from the inclusion body by denaturation and refolded to recover the activity of the immunotoxins due to the hydrophobicity nature of scFv [55, 56]. In contrast, nanobody-based immunotoxins can be purified in soluble format without denaturation and refolding, which greatly improves the efficiency of immunotoxin production and preserves the activity of toxins [53]. There are growing reports using nanobody-based immunotoxins to treat cancers by targeting a variety of antigens such as glypican-3, glypican-2, EGFR, HER2, VEGFR2, CD7 and CD38, which indicates the great potential of nanobody-based immunotoxins for cancer therapy [34, 57–59]. To the best of our knowledge, cancer therapy using CDH17 nanobody fused with toxin PE38 presented in this study was not previously reported. Kusano-Arai. O et al. had used full length monoclonal antibodies directed CDH17 to construct an immunotoxin cocktail through chemical conjugation with saporin toxin [20]. The immunotoxin cocktail was only tested the activity in vitro and the authors did not present any in vivo data. The tumor penetration might be a crucial issue for this full-length antibody-toxin due to its large molecule weight. In the present study, we fused CDH17 nanobody E8 with toxin PE38 to produce soluble immunotoxin without any denaturation and refolding procedures and obtained recombinant protein with high activity. This nanobody-based immunotoxin was extensively tested its activity in vitro and in vivo, showing superb anti-proliferation effect on CDH17-overexpressing gastric cancer cell lines, and significantly inhibits the tumor growth and prolongs the survival in CDH17-positive CDX and PDX models. PDX model is becoming a type of popular and reliable animal model for drug evaluation since it maintains the important characteristics of parental patient tumors, such as molecular phenotype, tumor heterogeneity, tumor microenvironment/structure and similar response to tested drugs [35, 60]. These strengths make PDX model more useful for prediction of therapeutic response than conventional CDX model. In the present study, the prominent anti-tumor effect of E8-PE38 immunotoxin on a CDH17-positive PDX model strongly supports the clinical translation for this novel therapeutic modality in GC therapy.

HER2 antibody (Trastuzumab and HER2-conjugated drug (T-DXd) have been approved for advanced HER2-positive gastric cancer [3]. But only 20% of gastric cancer patients highly express HER2 molecules [8], which limits the broad application of HER2 targeted therapy. It is difficult to compare the anti-tumor efficacy between HER2 antibody or conjugates with E8-PE38. Firstly, the expression levels of CDH17 and HER2 in the same gastric cancer cell line or cancer tissue are distinct. Second, the therapeutic modalities of CDH17 and HER2 targeting are involved in different mechanisms of action. In terms of MKN45 or TMK1 cells, they express higher level of CDH17 (Fig. 1) but lower level of HER2 [61, 62]. We have shown that 0.6 mg kg^− 1^ E8-PE38 immunotoxin could almost completely inhibit tumor growth induced by MKN45 cells in mice. However, 10 mg kg^−1^Trastuzumab just showed suboptimal tumor inhibition in HER2-overexpressing N87 cancer cell-induced mouse model [63]. Targeting CDH17 with nanobody for immunotoxin delivery might be a good complementary strategy for GC patients who are not suitable for HER2 targeted therapy since higher percentage (66%) of GC patients express CDH17 protein. On the other hand, chemotherapy is still a standard treatment for advanced GC. Fluoropyrimidines are major drugs for advanced GC chemotherapy. Combination cancer therapy is widely used in clinical settings and clinical trials to overcome tumor heterogeneity and development of drug resistance, at the same time, to obtain additive or synergistic tumor repression [64]. In the present study, we tested the combination effect of 5-FU and E8-PE38 on CDH17-postitive tumor control, and disclose that the combination treatment produces a better tumor repression than either drug alone, thus, indicating a novel therapeutic regimen for CDH17-positve GCs.

## Conclusions

In summary, two nanobodies were identified and their binding activities were extensively verified with protein, cells and in vivo tumor models in this study. The E8 nanobody as a lead could be used for the rapid imaging detection of CDH17-positive gastric cancer and highly efficiently deliver toxin PE38 into tumor tissues. The constructed E8-immunotoxin shows prominent anti-tumor effect in several gastric cancer models including PDX. Collectively, targeting CDH17 with nanobodies represents a new strategy for gastric cancer imaging and therapy. CDH17 nanobody immunotoxin is a novel and promising modality for targeted therapy in gastric cancer.

## Supplementary Information

**Additional file 1.** Supplementary figures, materials, and methods.

**Additional file 2:** **Supplementary table S1.**

## Data Availability

For data and materials requests, please contact the authors.
