# Supplementary material for: CDH17 nanobodies facilitate rapid imaging of gastric cancer and efficient delivery of immunotoxin
Source: Biomater Res. 2022 Nov 26;26:64. doi: 10.1186/s40824-022-00312-3 (PMC9701387; doi:10.1186/s40824-022-00312-3)
Supplement: Supplementary file 1 — Additional file 1. Supplementary figures, materials, and methods. [file 40824_2022_312_MOESM1_ESM.docx]

Supplementary Materials

CDH17 nanobodies facilitate rapid imaging of gastric cancer and efficient delivery of immunotoxin

Jingbo Ma^1,#^, Xiaolong Xu^1,#^, Chunjin Fu^1,#^, Peng Xia^1,2^, Ming Tian^1,2^, Liuhai Zheng^1^, Kun Chen^1^, Xiaolian Liu^3^, Yilei Li^4^, Le Yu^3^, Qinchang Zhu^5^, Yangyang Yu^6^, Rongrong Fan^7^, Haibo Jiang^8^, Zhifen Li^9^, Chuanbin Yang^1^, Chengchao Xu^1^, Ying Long^1*^, Jigang Wang^1*^, Zhijie Li^1*^

*Corresponding authors:

[li.zhijie@szhospital.com](mailto:li.zhijie@szhospital.com)(Zhijie Li);

[wangjigang@u.nus.edu](mailto:wangjigang@u.nus.edu)(Jigang Wang);

[13823730482@139.com](mailto:13823730482@139.com)(Ying Long).

^1^These authors contribute equally to this work and share the first-authorship.

^1^ The Second Clinical Medical College, Jinan University; The First Affiliated Hospital, Southern University of Science and Technology, Shenzhen, Guangdong 518020, P. R. China

^2^ Department of Hepatobiliary & Pancreatic Surgery, Zhongnan Hospital of Wuhan University, Wuhan, Hubei, 430072, P. R. China

^3^ Department of Pharmacy, Nanfang Hospital, Southern Medical University, Guangzhou 510515, P. R. China

^4^ Guangdong Provincial Key Laboratory of New Drug Screening, School of Pharmaceutical Sciences, Southern Medical University, Guangzhou 510515, P.R. China.

^5^College of Pharmacy, Shenzhen Technology University, Shenzhen, 518118, P.R. China.

^6^ Health Science Center, Shenzhen University, Shenzhen, Guangdong province, 518055, P. R. China

^7^Deapartment of Biosciences and Nutrition, Karolinska Institute. 14157 Stockholm.

^8^Department of Chemistry, The University of Hong Kong, Pok Fu Lam, Hong Kong, P. R. China.

^9^School of Chemistry and Chemical Engineering, Shanxi Datong University, Xing Yun Street, Pingcheng District, Datong, Shanxi Province 037009, P. R. China


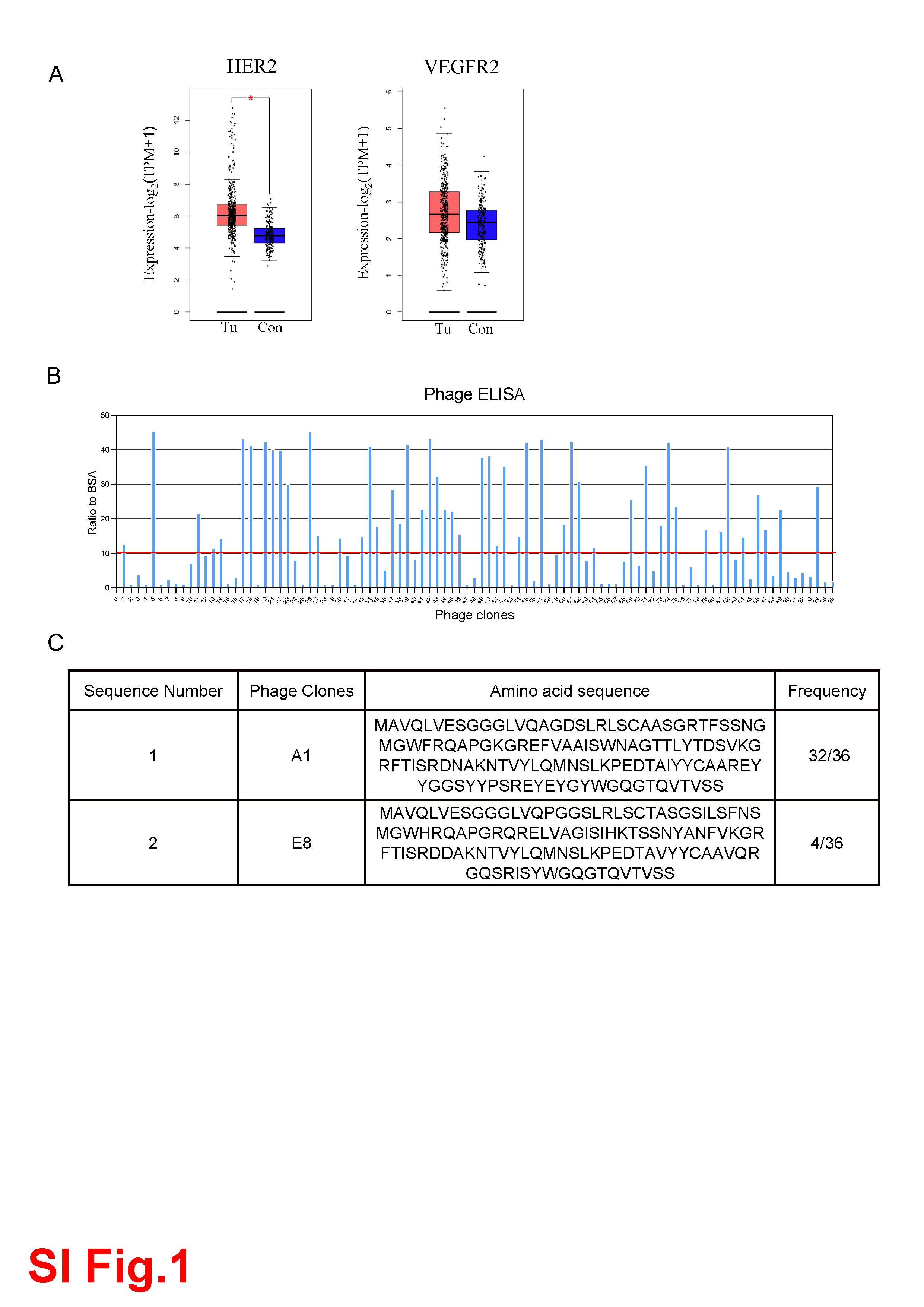


Fig. S1 a. RNA expression of HER2 and VEGFR2 (TPM, RNAseq) in gastric cancers and normal stomach controls. n=408(tumors) and 211(controls). b. Nanobody phage ELISA against CDH17. The absorbance ratio of CDH17 to control BSA ≥10 was considered positive clones. 50 potentially positive clones were identified. c. 36 nanobody clones were successfully sequenced. Two nanobodies were highly enriched. The frequency of A1 and E8 nanobodies was 89% (32/36) and 11% (4/36) respectively.


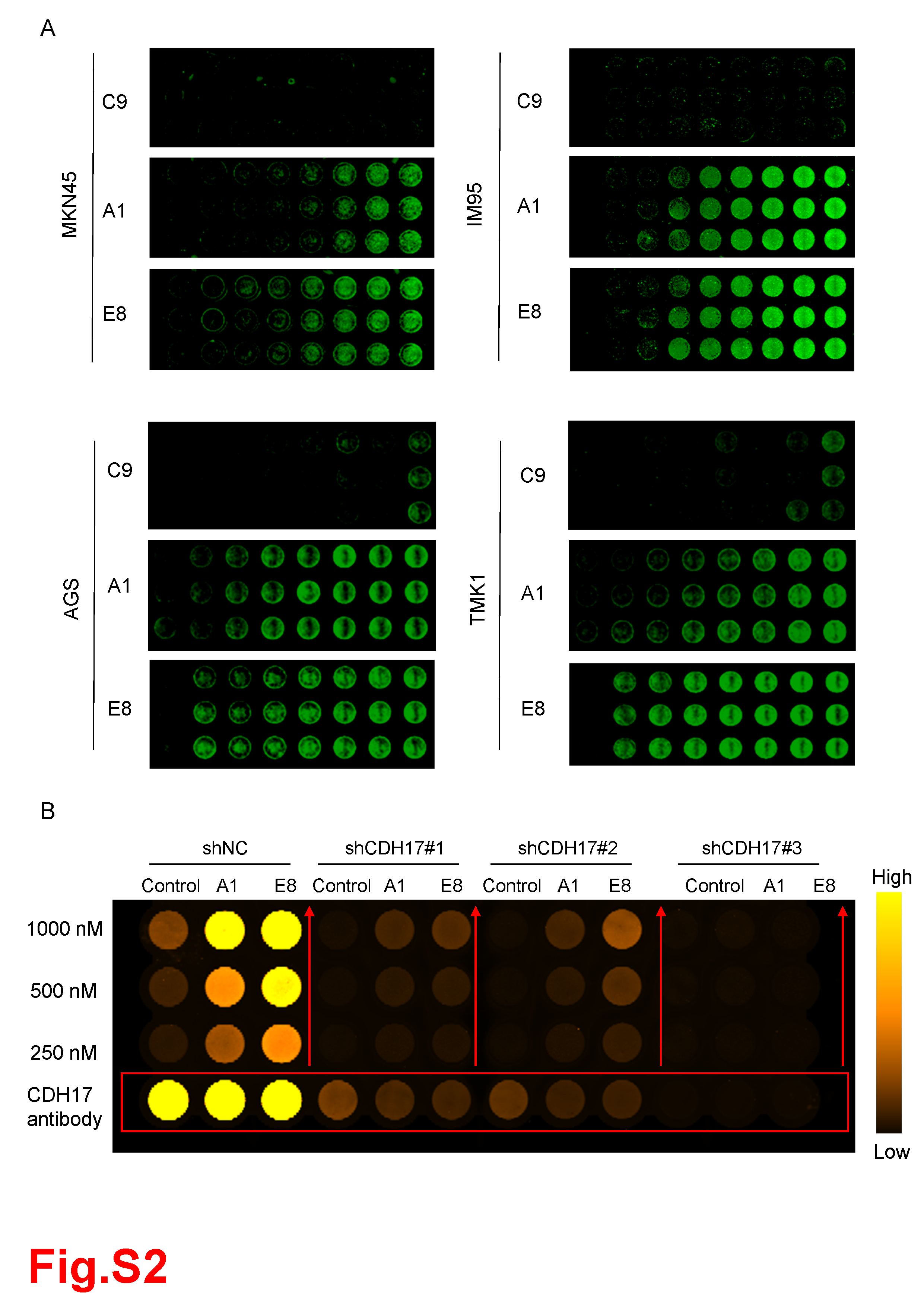


Fig. S2 a. Cell ELISA raw data related to Fig 2d. Con, A1 and E8 nanobodies were tested the binding activity to CDH17-positive cell lines MKN45, IM95, AGS and TMK1. Control nanobody did not bind to any CDH17-positive cell line. A1 and E8 nanobodies could target the CDH17-positive cells. b. Cell ELISA raw data related to Fig. 2e and 2f. Three shRNA constructs could significantly reduce the expression of CDH17 in MKN45 cells (bottom row) and shRNA#3 worked best. Knockdown CDH17 inhibited the binding activity of A1 and E8 nanobodies in MKN45 cells knocked down CDH17 with three shRNA constructs (top three rows).


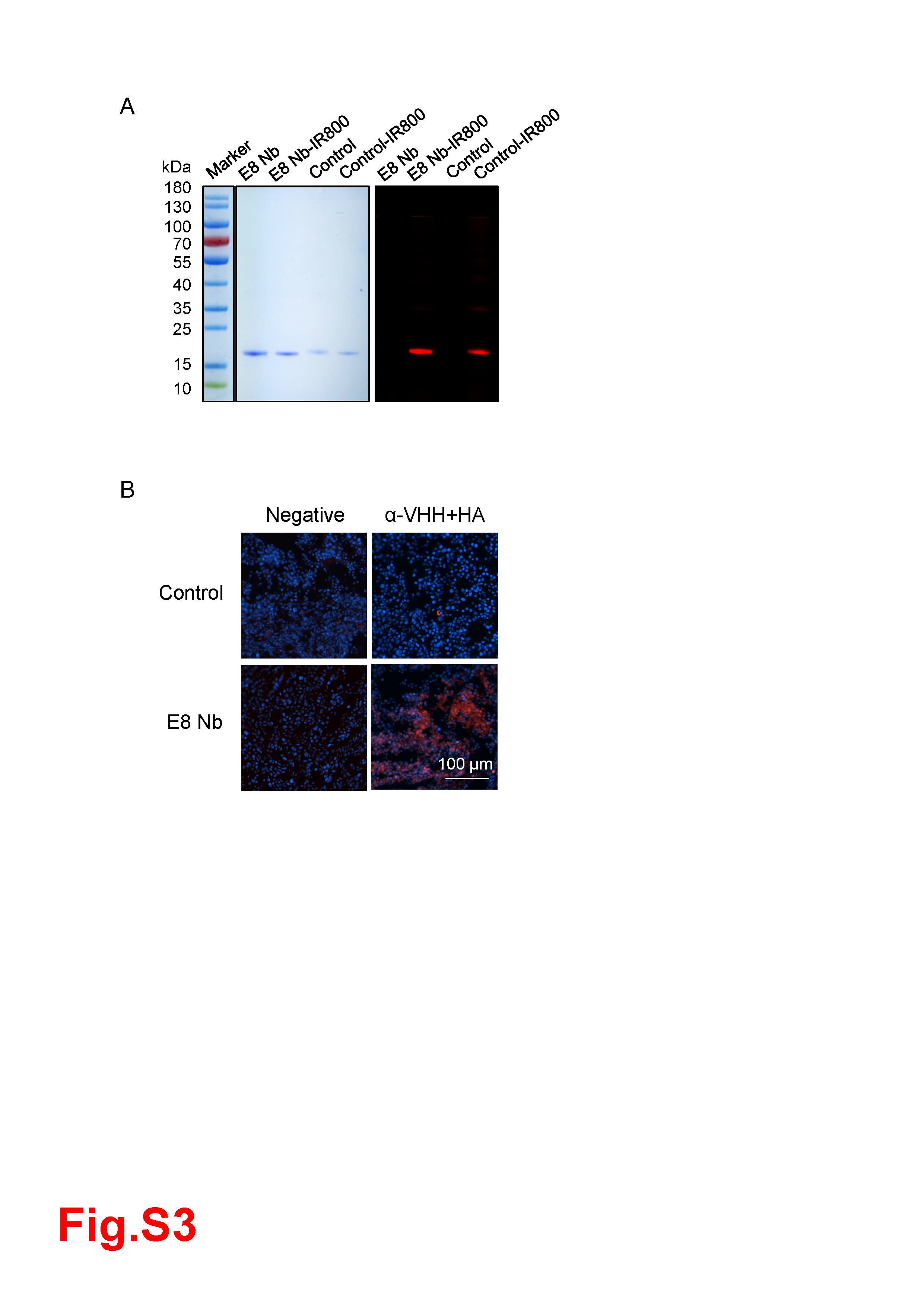


Fig. S3 a. SDS-PAGE analysis of nanobodies (left) and gel fluorescent scanning after labeling (right) with IR-800. b. Nanobody staining in tumor tissues received E8 or control nanobody injection. 100μg control nanobody or E8 nanobody was injected into MKN45-induced tumor bearing mice and allowed circulation for 12 hours. Tumor tissues were collected after perfusion and then applied for nanobody immunostaining with VHH plus HA antibodies. Control nanobody was not found in the tumor tissues. E8 nanobody can be detected specifically in tumor tissues. Scalebars:100μΜ.
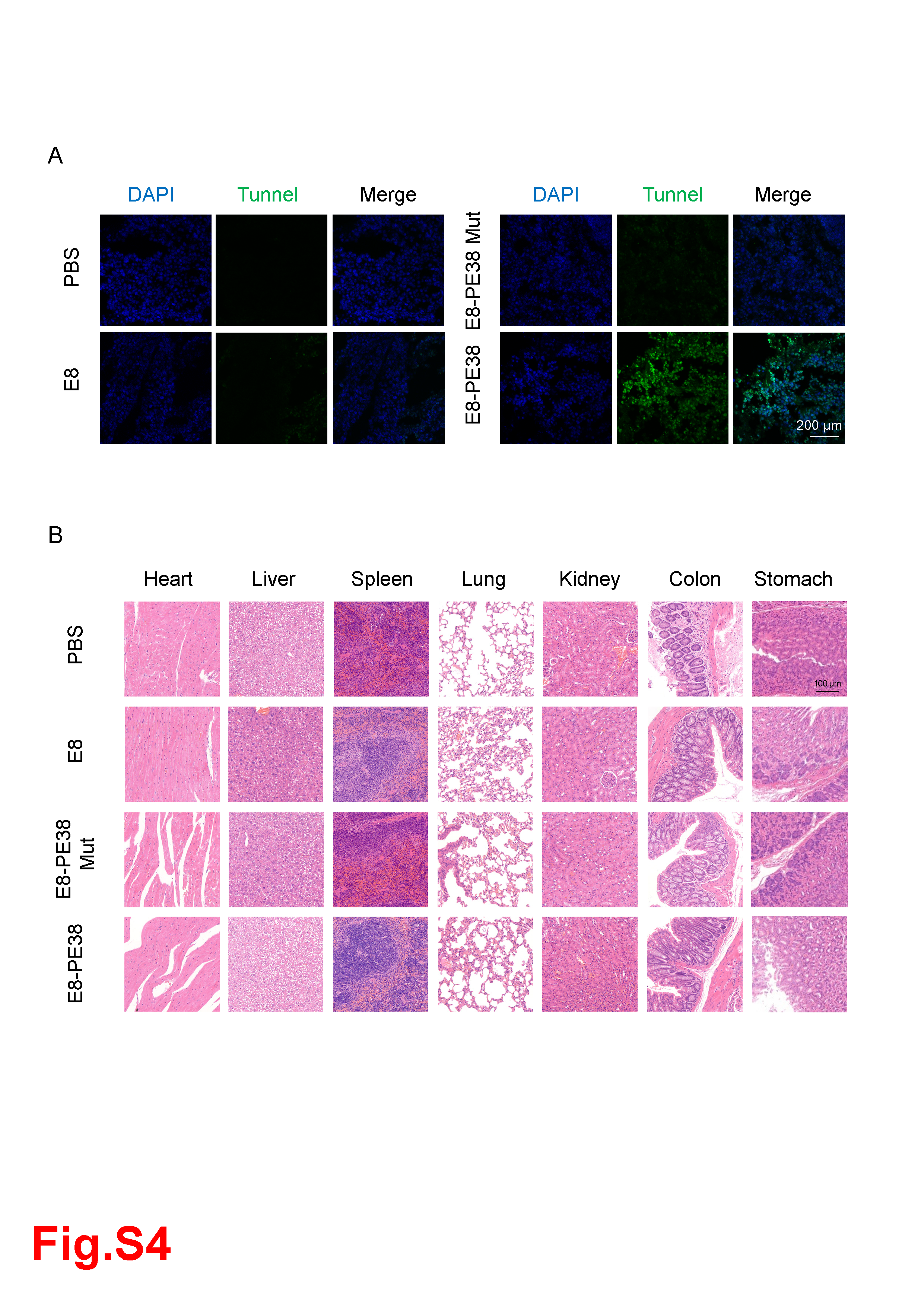


Fig. S4 a. TUNEL assay for mice treated with PBS, E8 nanobody, E8-PE38 and E8-PE38 immunotoxins. It was E8-PE38 only that can specifically induce tumor cell apoptosis in CDH17-positive tumor model. Scale bars: 200μM. b. H&E staining for major organs from mice treated in a. There was no visible change in terms of pathological analysis. Scale bars: 100μM.


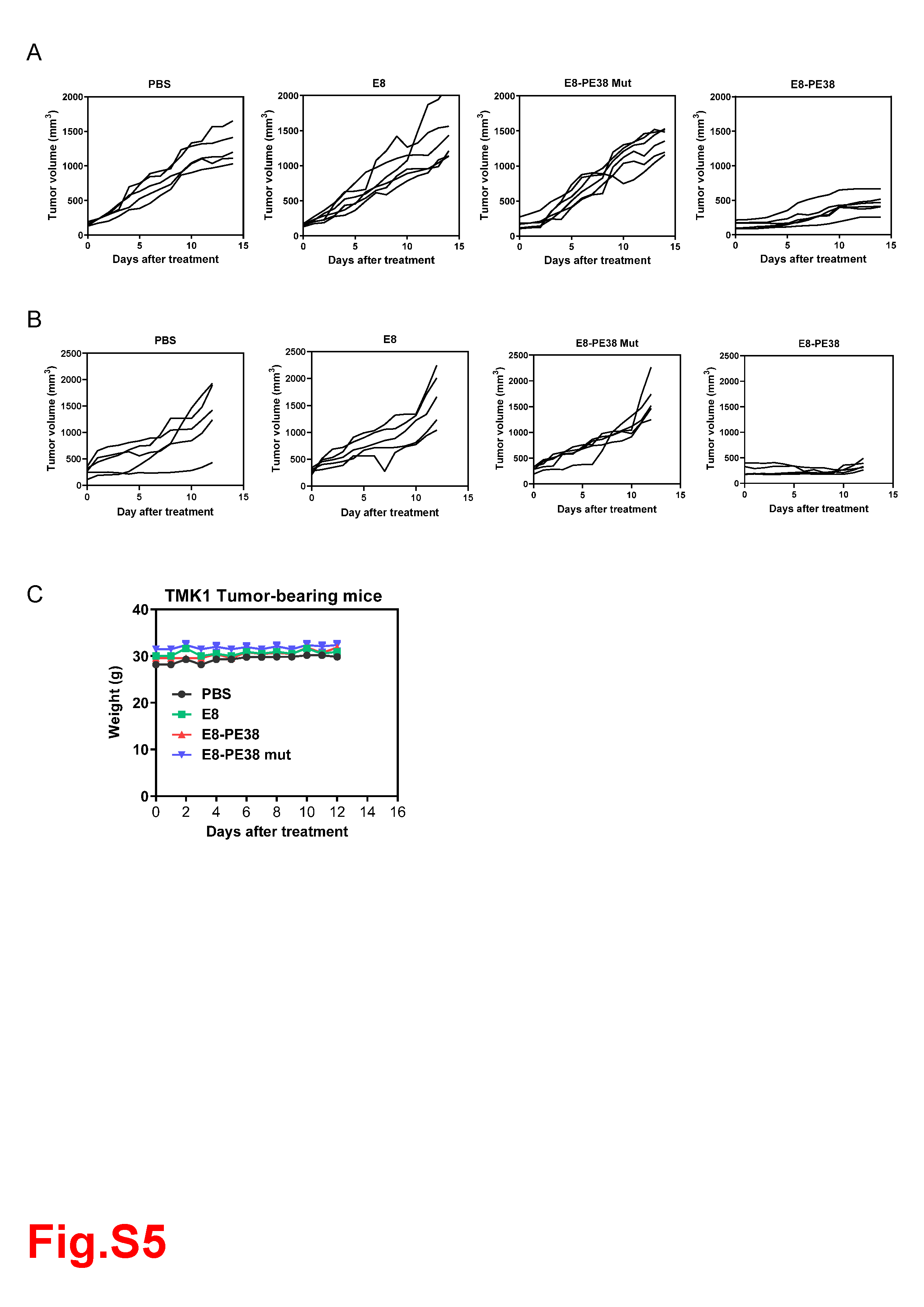


Fig S5 a. Individual tumor growth curves from MKN45 tumor-bearing mice treated with PBS, E8 nanobody alone, E8-PE38 mut and E8-PE38. b. Individual tumor growth curves from TMK1 tumor-bearing mice treated with PBS, E8 nanobody alone, E8-PE38 mut and E8-PE38. c. Body weight during the treatment from four groups in b.


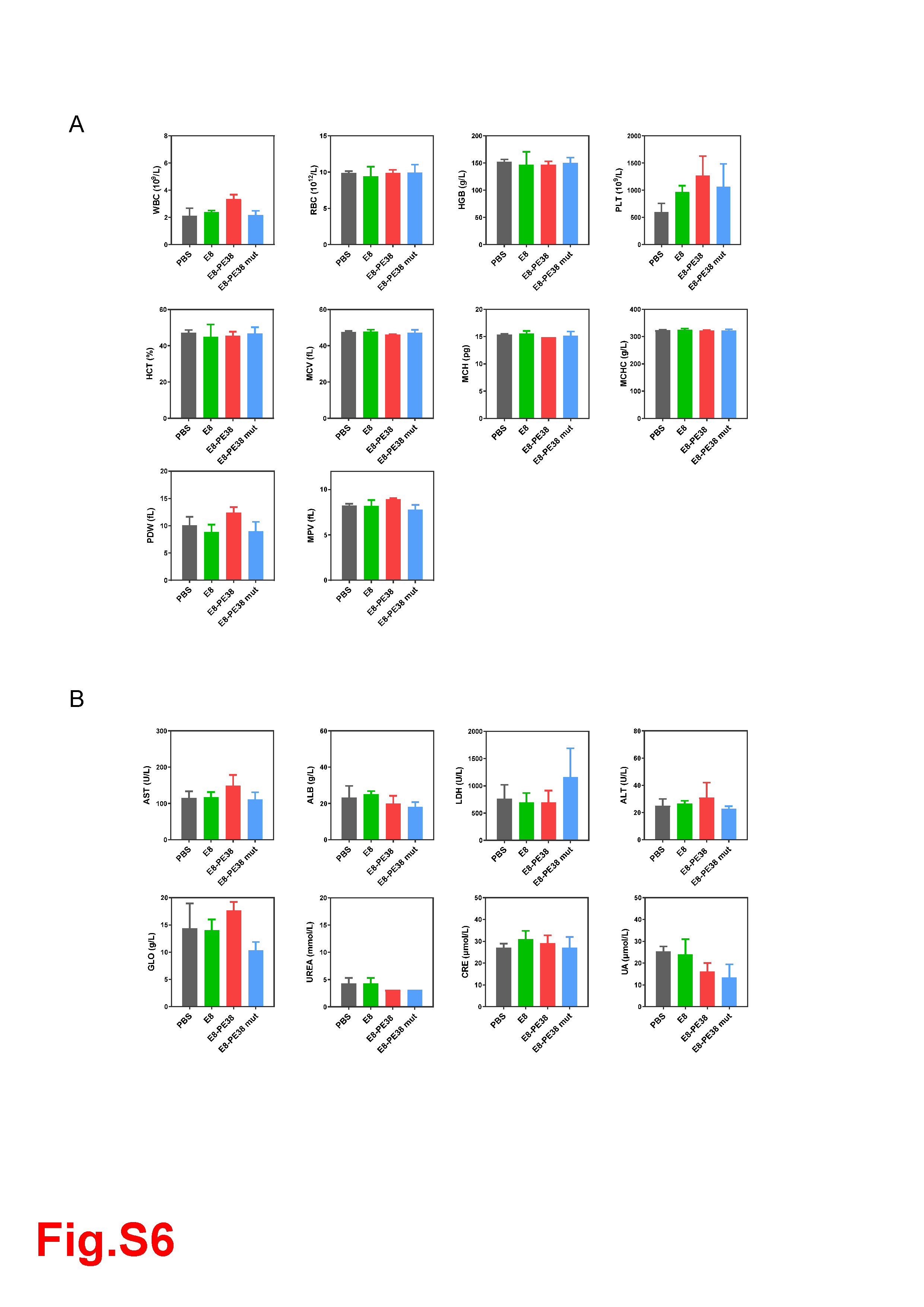


Fig. S6 a. The results of blood cell counting from treated mice in Fig. 5c. Various parameters from blood cell counting were not disclosed any significant alteration. b. Serum biochemistry analysis from treated mice in Fig. 5c. There was no significantly difference among the four groups received various treatments. Taken together, the data suggest that E8-PE38 immunotoxin is a safe modality for gastric cancer therapy. WBC, White blood cells; RBC, Red blood cells; HGB, Hemoglobin; HCT, Hematocrit; PLT, Platelets; MCV, Mean corpuscular volume; MCH, Mean corpuscular hemoglobin; MCHC, Mean corpuscular hemoglobin concentration; PDW, Platelet distribution width; MPV, Mean platelet volume; AST, aspartate aminotransferase; ALB. Albumin; LDH, Lactate Dehydrogenase; ALT, Alanine aminotransferase; GLO, Globulin; CRE, Creatinine; UA, Uric acid.


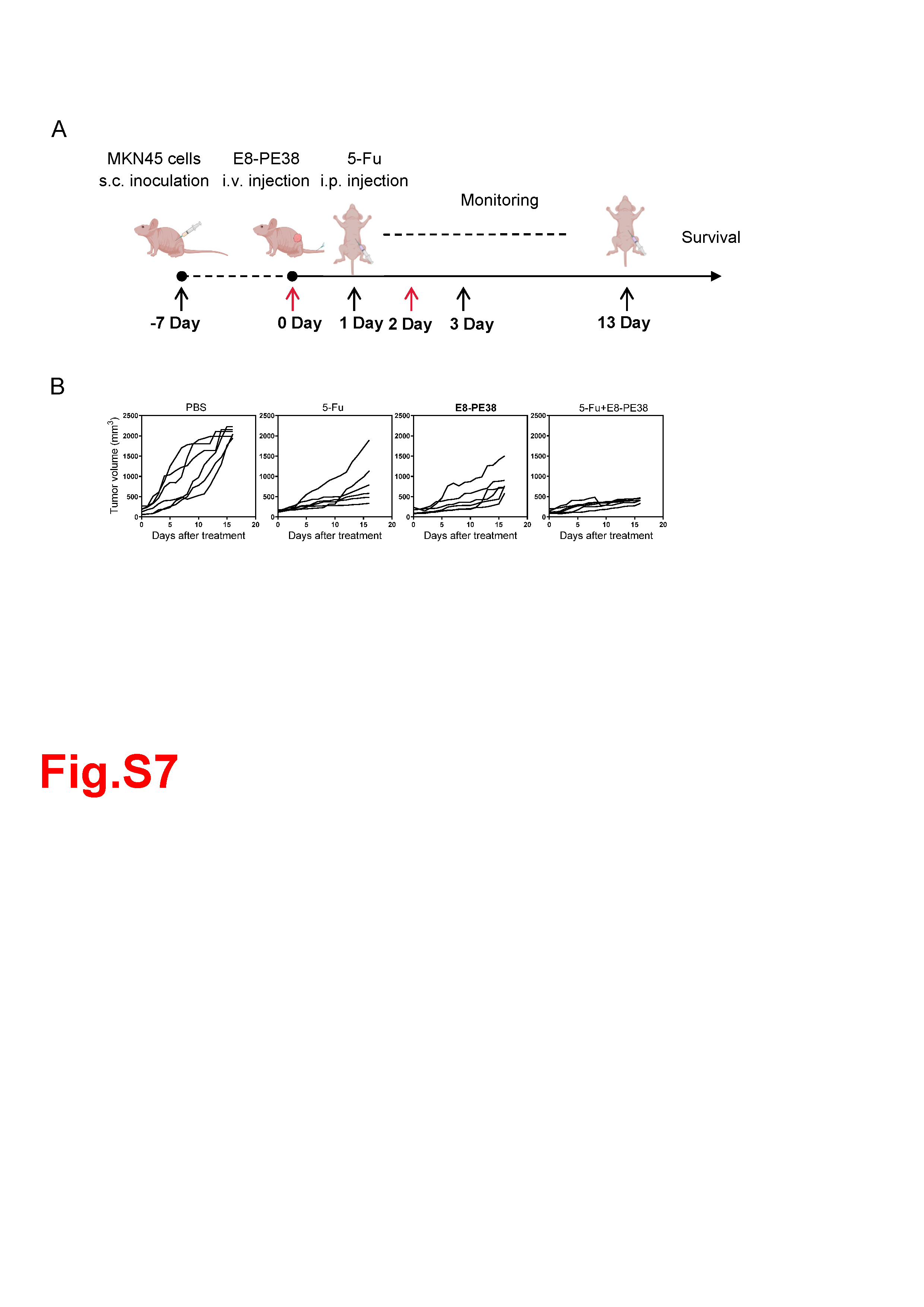


Fig. S7 a Schema of animal treatment schedule for combination therapy with 5-FU and E8-PE38. A total of seven injections for each drug were given MKN45 tumor-bearing mice every other day within 14 days. b Individual tumor growth curves from MKN45 tumor-bearing mice treated with PBS, 5-FU, E8-PE38 and 5-FU+E8-PE38 in Fig. 5J.

Table S1 Clinical information for microarray samples


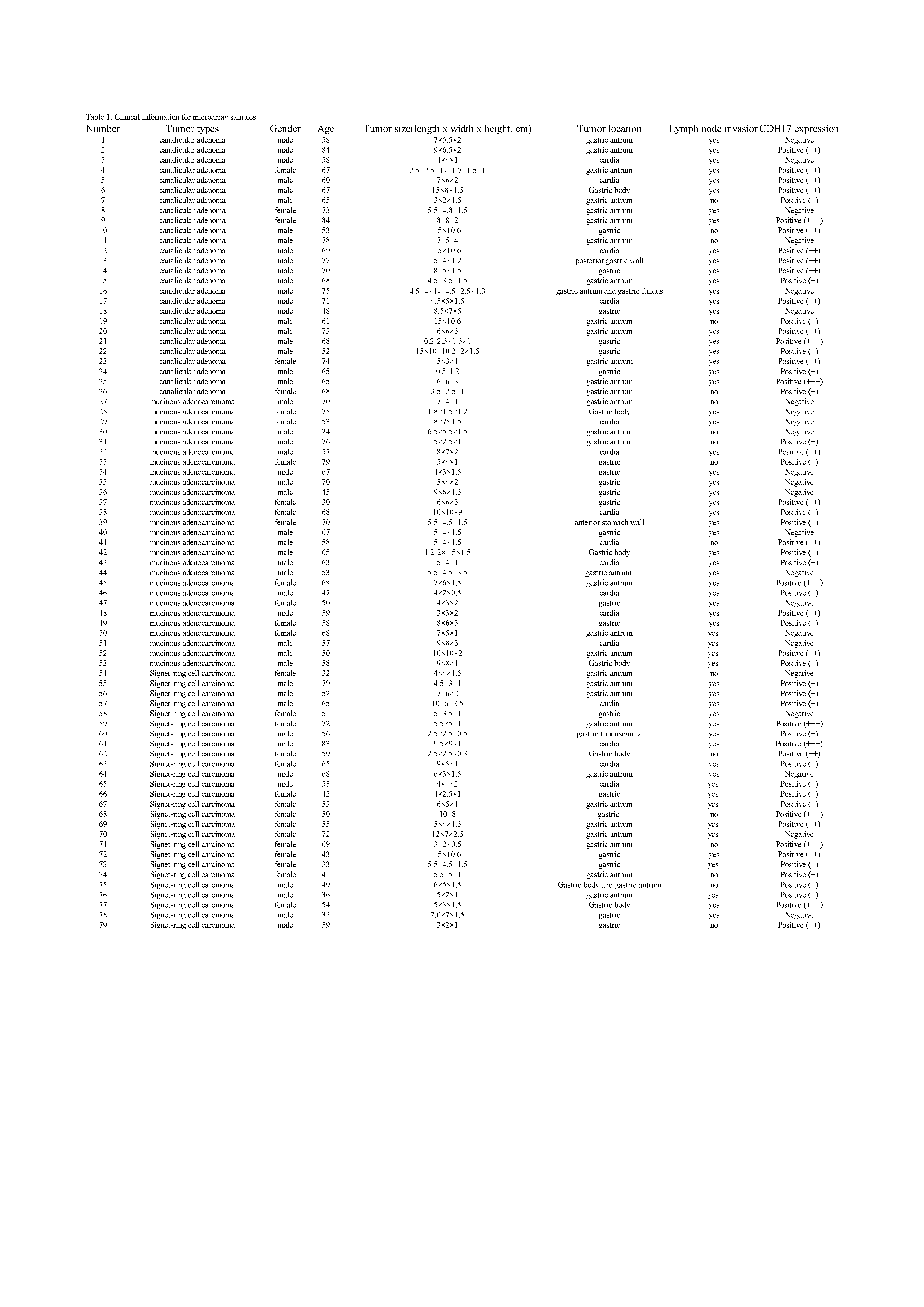


**Supporting Materials and Methods**

**Analysis of CDH17 RNA expression in gastric cancer**

mRNA expression for CDH17, HER2 and VEGFR2 in gastric cancer and normal stomach was collected in GEPIA (http://gepia.cancer-pku.cn/) database. The number for tumors and normal controls is 408 and 211 respectively

**Plasmid construction**

Sequences encoding domain 1-3 of human and mouse CDH17, nanobodies (control, A1 and E8), E8-PE38 and E8-PE38 mut were optimized for E.coli expression system and synthesized by GENEWIZ and cloned into vector pET-14B with NdeI and BamHI sites. The nanobody sequences were attached with a HA tag for detection and a cysteine amino acid at C-terminal for further modification.

**Nanobody ELISA**

To analyze the binding activity of nanobodies to CDH17 domain1-3, ELISA was performed. In brief, the purified human CDH17 domain1-3 was coated on 96-well microplates (10 μg/ml) and incubated at 4 °C overnight, followed by the incubation with 3% BSA-PBS solution for 1 h at RT. The nanobodies were diluted from 4000 pm to 62.5 pm with 1×PBST and incubated in plates at RT for 1 h. The plates were then washed three times with PBST (PBS+0.1% Tween20) and nanobodies were detected by an HRP-conjugated anti-HA tag antibody (SinoBiological, China) and TMB peroxidase substrate (BioLegend, San Diego, CA, USA). The absorbance was then measured at 450 nm.

**Affinity measurement**

To determine the binding affinity of A1 and E8 nanobody to CDH17, an SPR assay was performed on Biacore T100 (GE Healthcare) according to the manufacturer’s instructions. The CM5 sensor chip (GE Healthcare), was used for capturing purified 1-3 domain of human CDH17 protein at a concentration of about 10μg/mL and a flow rate of 10 μl/min for 1 min. This resulted in coupling of ~300 RU CDH17domain1-3 to the sensor chip surface. Meanwhile, one flow cell of the sensor chip was left without captured CDH17 domain 1-3 to provide a reference control. Nanobodies were prepared at different concentrations starting from 2000 nM to 7.81 nM using a 2-fold serial dilution in running buffer (1×PBS, 0.005% Tween-20, pH 7.4).

For each nanobody, sensorgrams were recorded for different analyte concentrations at a flow rate of 30 µL/min and a data collection rate of 1 Hz. Analyte injections were performed with association and dissociation phases of 180 s and 300 s, respectively. Prior to data analysis, reference and zero concentration data were subtracted from the sensorgrams. The collected data were fitted according to a 1:1 Langmuir binding model (one Nb to one monomer of CDH17 domain 1-3) from the experimental flow cells of a single biosensor chip. All experimental data were treated with Biacore T100 Evaluation Software to calculate the kinetic K_on_ and K_off_ rates and the equilibrium dissociation constant (K_D_).

**Lentivirus packaging and establishing stable cell line**

pLVX-shRNA vector containing CDH17 shRNAs or control shRNA was co-transfected with pMD2.G and psPAX2 into 293-T17 cells. The supernatants at 48 h and 72 h post-transfection were collected and concentrated. The lentivirus products were used to transduce MKN45 and IM95 cells and stable cell lines were selected by 2 μg/mL puromycin. The sequences for shRNAs were listed in the table below.

T

Table S2, shRNA sequences used in this study

| CDH17 shRNA#1 | 5’-GCATTGTTTCTTTACCAGTTA-3’ |
| --- | --- |
| CDH17 shRNA#2 | 5’-GCAGTTGTGTTTATCCGCATA-3’ |
| CDH17 shRNA#3 | 5’-CCAGTCCCTATCACCATAGAA-3’ |
| Control shRNA | 5’- GCAAGCTGACCCTGAAGTTTA-3’ |

**Imaging of zebrafish embryos with nanobody liposomes**

The study with zebrafish embryos in this project was also approved by Institutional Animal Care and Use Committee (IACUC) of the Shenzhen People’s hospital. Wild-type AB zebrafish were obtained from China Zebrafish Resource Center (Wuhan, China). Zebrafish embryos were raised at 28℃ under standard conditions. Zebrafish embryos at 48 h post-fertilization (hpf) were dechorionated prior to cell injection. MKN45 cells were fluorescently labeled by 5 µM CM-DiI (Thermo Fisher Scientific) for 20 min at 37℃. CM-DiI-labeled cells were further incubated with liposome-FITC labeled with E8 or Con nanobody at room temperature for 30 min. Approximately 100 cells were then injected into common cardinal vein near the embryo’s heart using a micropipette and pump (World Precision Instruments). Engrafted zebrafish embryos were examined and photographed using an Olympus MVX10 Zoom Fluorescence Macro System Microscope within one hour after injection. Ten embryos of each treatment were examined.

**Western blot**

Briefly, concentrations of purified nanobodies were determined by BCA kit (Pierce). And 2 μg of nanobodies was mixed with loading buffer for SDS-PAGE analysis. The proteins were transferred to PVDF membranes (Millipore, USA) at a constant current of 200 mA for 30 min. The membranes were blocked with 5% skimmed milk-TBS solution and then incubated with mouse anti-HA tag antibody or mouse anti-6 x His tag antibody in TBST (TBS+0.1% Tween20) at 4℃ overnight. The membranes were washed with TBST and incubated with HRP-conjugated goat anti-mouse IgG Antibody. The membranes were detected by enhanced chemiluminescence.

To detect CDH17 expression levels in gastric cell lines, TMK1, AGS, MKN45 and IM95 were cultured in 6-well plates, and when the cell confluence reaches 80%, the cells were washed with PBS and lysed by adding 200 μl of RIPA lysis buffer per well. The cell lysate was analyzed protein concentration by BCA kit (Pierce). 30 μg total proteins were mixed with loading buffer for SDS-PAGE analysis and conducted the membrane transferring and blotting as described above.

**Tissue distribution of nanobodies**

When tumors grew to approximately 500 mm^3^, the MKN45 tumor-bearing mice were randomly divided into two groups (n=3) and injected intravenously 100 μg E8 Nb-IR800 and C9 Nb-IR800 respectively. 12h after injection, the mice were sacrificed, and major organs (heart, liver, spleen, lung, kidney, stomach, colon) and tumor were harvested for immunofluorescence and immunohistochemistry.

For immunofluorescence staining of tumor tissue, 10 µm frozen tissue sections were incubated with mixed primary antibodies (rabbit anti-HA, (CST, USA) and rabbit anti-VHH, (Genscript, China)) overnight at 4℃. The slides were washed three times with TBST and incubated with secondary anti-rabbit AF594 antibody for 1h at RT. The immunofluorescence signal was analyzed by laser scanning confocal microscope (Leica, Germany).

For immunohistochemistry staining of tissues, 10 µm frozen sections were incubated with mixed primary antibodies (anti-HA and anti-VHH) overnight at 4°C. The slides were washed and incubated with secondary biotinylated anti-rabbit antibody (Thermo Scientific, USA). After the incubation for 1 h at RT, the slides were incubated with ABC solution (Vector, USA) for 1h at RT. The color development was performed using DAB solution. The images were obtained using an inverted light microscope (Leica, Wetzlar, Germany).

**Toxicological analysis**

Blood samples and major organs were collected from the mice treated in Fig 5c when mice were euthanized. Samples were processed for blood cell counting, serum biochemistry analysis. Various organs were fixed and embedded in paraffin. H&E staining was performed for pathological analysis.
